# Exploiting Avidity Effects for the Discovery of Low Affinity Protein-Binding Fragments

**DOI:** 10.1021/acs.jmedchem.5c01742

**Published:** 2025-09-15

**Authors:** Isuru M. Jayalath, Donella Beckwith, Jihyeon Yoon, Xingui Liu, Thomas Kodadek

**Affiliations:** † Department of Chemistry, 145764 The Herbert Wertheim UF Scripps Institute for Biomedical Innovation & Technology, 120 Scripps Way, Jupiter, Florida 33458, United States; ‡ Deluge Biotechnologies, 6671 W. Indiantown Rd., Suite 50-325, Jupiter, Florida 33458, United States; § Department of Medicinal Chemistry, College of Pharmacy, 3463 University of Florida, Gainesville, Florida 32610, United States

## Abstract

Fragment-based drug discovery (FBDD) is a powerful approach to the development of pharmaceuticals and probe molecules and there is broad interest in the development of new platforms for their discovery. Here, we introduce a workflow in which low molecular weight organic molecules displayed on the surface of TentaGel beads are exposed to a multimeric, fluorescently labeled target protein. Using tetrameric or dimeric protein targets, we show that beads that display even weak ligands (*K*_D_s in the high μM to low mM range) stably capture the protein due to avidity effects, thus allowing a simple “pull-down” protocol to be employed for fragment discovery. We also demonstrate that the platform is capable of supporting a “fragment growth” screen, which is a typical strategy to advance a fragment to a higher-affinity lead molecule. This platform is inexpensive and requires no specialized infrastructure.

## Graphical abstract

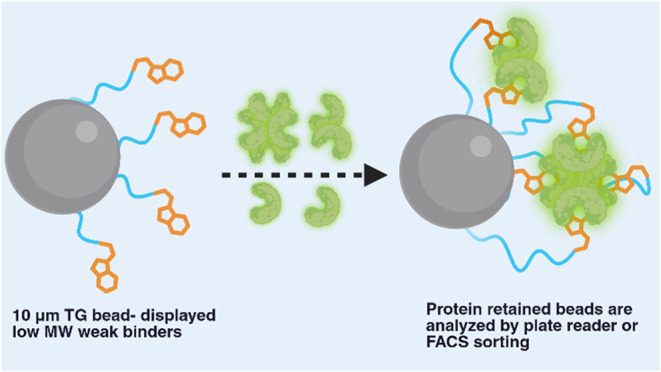

## Introduction

Fragment-based drug development (FBDD) has emerged as a powerful and effective method for the creation of new pharmaceuticals and probe molecules. The first step in this workflow is the discovery of low molecular weight (<300 Da) ligands for the protein of interest, providing a highly atom-efficient starting point for lead development.^1–4^ To date, this approach has led to the development of eight approved drugs^5–12^ plus a substantial number of clinical candidates.^1^ FBDD offers several advantages over traditional high-throughput screening (HTS), including the ability to explore a larger chemical space with smaller libraries,^13^ ready applicability to targets lacking enzymatic activity,^4,14^ and the possibility of applying fragment hit rates as a measure of target druggability.^4,15^

Because of their small size, fragments usually bind to proteins with low affinity (*K*_D_s typically in the high μM to low mM range). Because of this, binding assays that require a washing step, thus taking the system out of equilibrium, are not suitable for fragment discovery. The complexes are simply too labile. Thus, the discovery of protein-binding fragments currently relies heavily on biophysical techniques capable of assaying binding under equilibrium conditions, including X-ray Crystallography,^16^ Nuclear Magnetic Resonance (NMR) spectroscopy,^17,18^ Surface Plasmon Resonance (SPR),^19^ Isothermal Titration Calorimetry (ITC),^20^ Differential Scanning Fluorimetry (DSF),^21^ and others.^2,12^

These methods have various advantages and limitations.^12^ The most powerful existing methods are probably NMR and SPR. The most sophisticated SPR instruments are capable of measuring on and off rates of small molecules to an immobilized protein in 1536-plex format, allowing large numbers of compounds to be analyzed for binding in a few days. NMR-based methods similarly are also capable of investigating thousands of small molecule-protein interactions given the appropriate infrastructure for semiautomated handling. NMR has the additional advantage of immediately providing structural data on the fragment-protein complex, which is not the case for SPR-driven discovery. Some limitations of these techniques are the requirement for expensive and specialized instrumentation and, in the case of NMR, the requirement for large quantities of protein, which, for some experiment, must be uniformly ^15^N-labeled.^17,18^ Finally, only fragments that exhibit good solubility in aqueous solutions can be used in these screens since they must be present at high micromolar to millimolar concentrations.^4,22,23^ Thus, there remains a need for the discovery of protein-binding fragments, particularly protocols that could be carried out inexpensively using equipment accessible to almost all chemistry and biochemistry laboratories.

We report here a “low tech” “pull-down” assay for the analysis of protein–ligand interactions that is capable of registering low affinity complexes in the high μM-low mM range. As mentioned above, this type of experiment is not generally useful for capturing weak complexes. However, it has been demonstrated previously that the association of multimeric proteins with ligands displayed on a surface can result in quite stable, long-lived complexes thanks to avidity effects (Figure 1). Specifically, earlier studies demonstrated that dimeric or higher order homo-oligomeric proteins interact with TentaGel-displayed ligands with apparent affinities 100 times or more better than that observed in solution.^24–31^ Based on these observations, we hypothesized that multivalent binding between bead-displayed fragments and an oligomeric protein might stabilize these weak complexes to the point that they would survive being taken out of equilibrium during the washing and analysis steps. Using Streptavidin (SA), a native tetramer,^32,33^ as a model target protein, we show that this is indeed the case and that libraries of bead-displayed small molecules can be screened using this technology inexpensively, with relatively high throughput, low protein consumption, and without the requirement for sophisticated infrastructure. To generalize this workflow to proteins that are not native oligomers, we also evaluate whether low affinity fragments can be identified that bind a fusion protein comprised of Glutathione-S-Transferase (GST), a native homodimer, and a monomeric protein of interest (POI). Indeed, we show that this is the case and report fragments that engage the PRU domain of Rpn13, one of the Ubiquitin receptors of the 26S proteasome.

**Figure 1:**
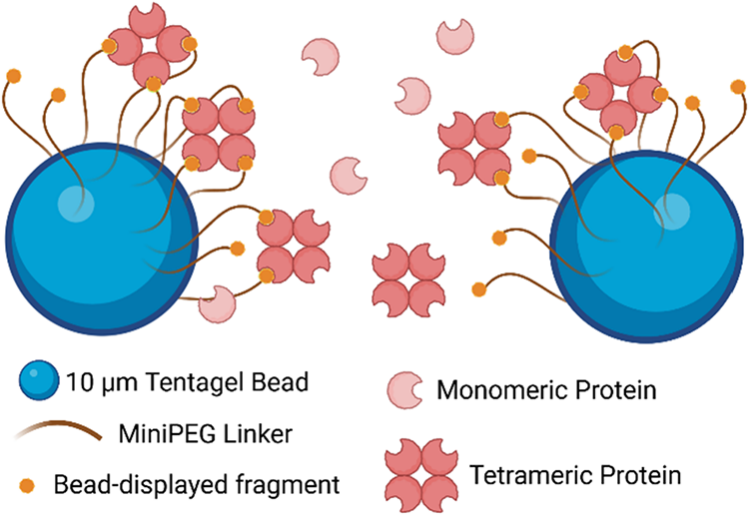
Schematic representation of the bead-based fragment screening method. The orange circle represents a fragment, and the light red “Pac-Man” shape represents the target protein (TP). Simultaneous engagement of multiple fragments with the multimeric TP results in a stable complex.

## Results

### Avidity-Driven Stabilization of Weak Complexes on Resin

The hypothesis we wished to explore is whether a low affinity, small molecule ligand, when displayed on the surface of a TentaGel bead, could stably capture a multimeric protein through multivalent, avidity-driven binding. A useful model system for this investigation was suggested by the results of a screen of a DNA-encoded library (DEL) of nonpeptidic macrocycles against fluorescently labeled SA.^34^ 28 *bona fide* ligands were identified in this screen, all of which contained the meta-substituted pyridine side chain highlighted in Figure 2a. Twenty-four of the 28 hits also included the main chain thiazole unit (also highlighted in Figure 2a), and the remaining four ligands contained a closely related oxazole unit at this position. This suggested these two units together constituted the core pharmacophore. This hypothesis was confirmed by showing replacement of the pyridine with a phenyl ring, or the thiazole with an isoxazole, almost completely abolished binding.^33^

**Figure 2:**
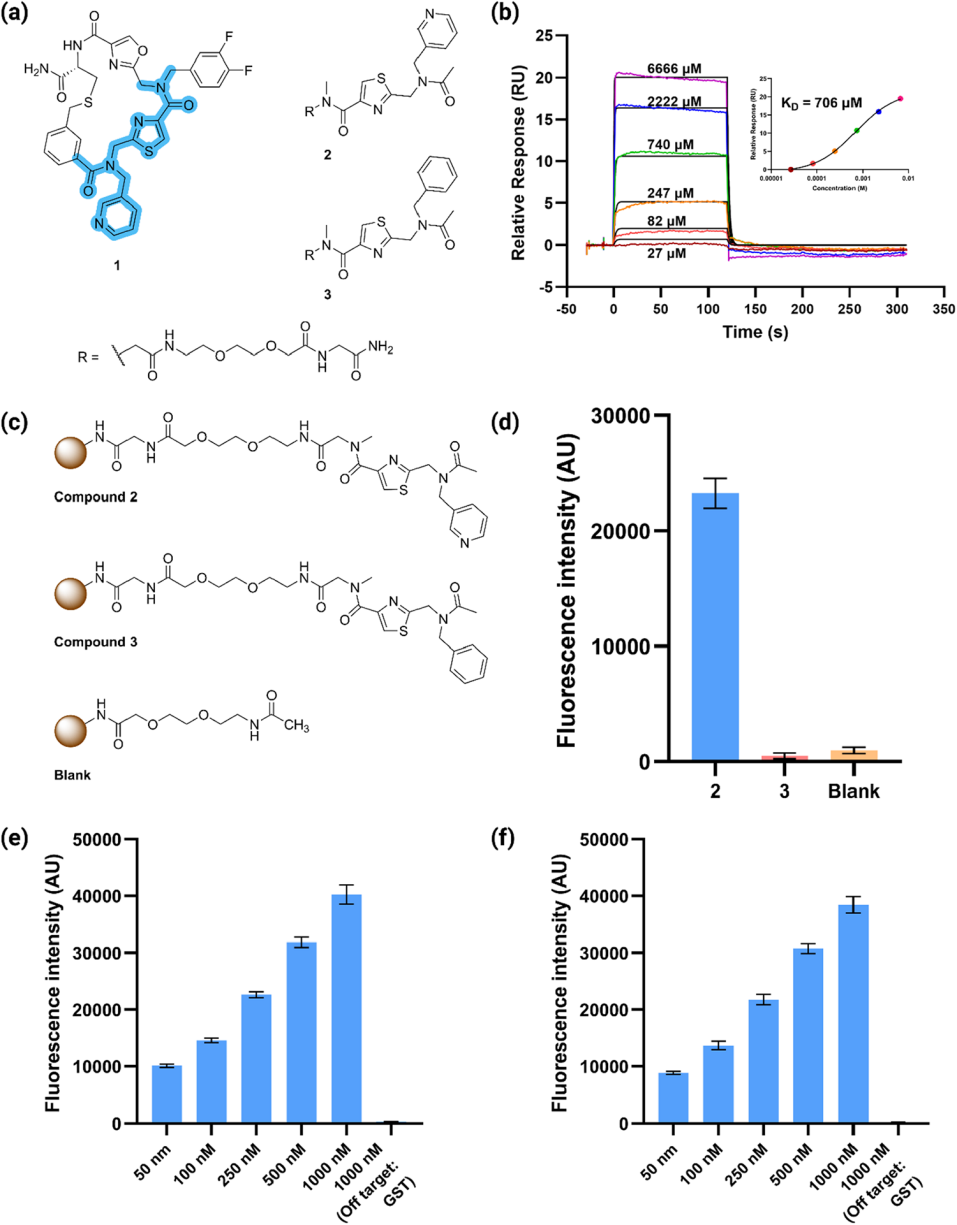
Identification of a weak streptavidin binder. (a) Structures of the SA-binding macrocycle **1** and its derived weak binders: compounds **2** and **3**. (b) SPR sensograms of compound **2** binding to immobilized SA (inset shows the affinity fitting). (c) Structures of the bead-displayed compounds and blank control. (d) Fluorescence intensity of bead-displaying indicated compound after incubation with SA-647 (500 nM) for 1 h in PBST at room temperature. (e) Fluorescence intensity of 10 μm TentaGel beads displaying compound **2** after incubation with the indicated concentrations of Alexafluor 647-labeled SA (SA-A647) for 1 h in PBST at room temperature. The final bar represents an off-target, glutathione S-transferase (GST). (f) Same as (e), except the beads were washed thoroughly, then suspended in buffer lacking protein for 24 h, demonstrating the high level of kinetic stability of the on-resin complex. In (d–f), the bars represent the average of three technical replicates, each of which employed 250,000 beads.

This led us to wonder if the thiazole-pyridine portion of the molecule might bind independently to SA and, if so, would exhibit a low affinity typical of a fragment, allowing us to readily test the hypothesis mentioned above. Thus, we synthesized this compound with a “mini-PEG” unit attached to the C-terminus to enhance solubility (compound **2**, Figure 2a). Surface plasmon resonance (SPR) measurements using immobilized SA and soluble **2** confirmed binding and revealed a *K*_D_ of 706 μM (Figure 2b), which is typical for a protein-binding fragment. Compound **3**, in which the critical pyridine ring was replaced with a benzene unit, showed no detectable binding to SA (Figure S1), providing an important negative control.

Compounds **2** and **3** were then constructed on 10 μm TentaGel beads via straightforward solid-phase synthesis, along with acetylated beads, which as a “blank” (Figure 2c). TentaGel beads consist of an amino-polystyrene core onto which is grafted a thick layer of amine-terminated PEG chains. An additional mini-PEG spacer was incorporated prior to coupling the compounds to further improve spatial accessibility and aqueous display of the compounds (see Experimental Section for detailed synthesis). The beads were incubated with AlexaFluor 647-conjugated SA (SA-647) in phosphate-buffered saline supplemented with 0.05% Tween 20 (PBST) for 1 h at room temperature in the dark. Following incubation, the beads were washed thoroughly (see Experimental Section for a detailed protocol), then resuspended in PBST. The amount of fluorescent SA retained by the resin was analyzed using a Tecan multiwell plate reader equipped with appropriate excitation and emission filters (excitation at 650 nm, emission at 665 nm).

As shown in Figure 2d, binding of SA-647 to bead-displayed **2** was readily detectable, while beads displaying compound **3** failed to retain more labeled SA than the acetylated “blank” beads. To further assess the stability of the on-resin **2**·SA complex, we repeated the experiment across a range of SA-647 concentrations. The fluorescence signal increased proportionally with SA concentration (Figure 2e). Remarkably, even at SA-647 concentration of 50 nM, a concentration about 14,000-fold below the intrinsic *K*_D_ of the complex, above background binding was readily detectable.

To evaluate binding selectivity, bead-displayed compound **2** was incubated with stained glutathione S-transferase (GST) (1 μM) as an off-target control (the degree of binding was visualized by staining with a labeled antibody). No detectable signal was observed (Figure 2e), confirming that compound **2** interacts selectively with SA.

To determine if ligand **2** interacts with the biotin-binding surface of SA, we preincubated SA-647 with excess biotin and repeated the binding assay. This ablated retention of SA-647 by resin-displayed **2**, suggesting it indeed engages the biotin-binding surface of SA (Figure S2).

To assess the kinetic stability of the complex, beads displaying compound **2** were incubated with SA-647, the beads were washed thoroughly, then resuspended in PBST, lacking additional protein, for 24 h. The fluorescence signal intensity was then measured, as shown in Figure 2f. The results were almost identical to those obtained in Figure 2e, demonstrating that the on-resin **2**·SA complex is remarkably stable over a period of many hours.

These results suggest that this simple pull-down assay could support a screen of bead-displayed weak-binding fragments against a multimeric protein. With this in mind, we proceeded to evaluate the robustness of this method using statistical metrics commonly applied in high-throughput screening, such as the *Z*′ factor.^35^ *Z*′ factor was determined from multiple replicates of an experiment in which bead-displayed compound **2** or compound **3** were incubated with SA-647 (500 nM) in PBST as described above (see Experimental Section for details). The amount of fluorescent signal retained on the beads after thorough washing was measured using a plate reader. The calculated *Z*′ factor for the assay was 0.66, indicating excellent assay quality and suitability for high-throughput fragment screening applications.

### The Stability of the On-Resin Complex Is Dependent on Multivalent Binding

Is this remarkably stable binding of SA to bead-displayed **2** indeed the result of multivalent interactions? To investigate this, compound **2**-displaying, compound **3**-displaying, or blank (acetylated) beads were incubated with tetrameric SA-647 or iFluor647-conjugated monomeric streptavidin (mSA-647). Biotin-displaying beads (Figure S3) were also included in this experiment as a high-affinity comparator. As expected, beads displaying compound **2** displayed strong binding to tetrameric SA-647. In contrast, beads displaying compound **2** did not retain detectable levels of mSA-647 (Figure 3a). Conversely, binding of the monomer to biotin-displaying beads was readily observable, albeit at a reduced intensity relative to the tetramer. No binding of either protein to compound **3**-displaying beads was observed. These data are consistent with the idea that compound **2** depends heavily on avidity effects to retain tetrameric SA, while the intrinsically high-affinity biotin ligand is much less dependent on multivalency.

**Figure 3:**
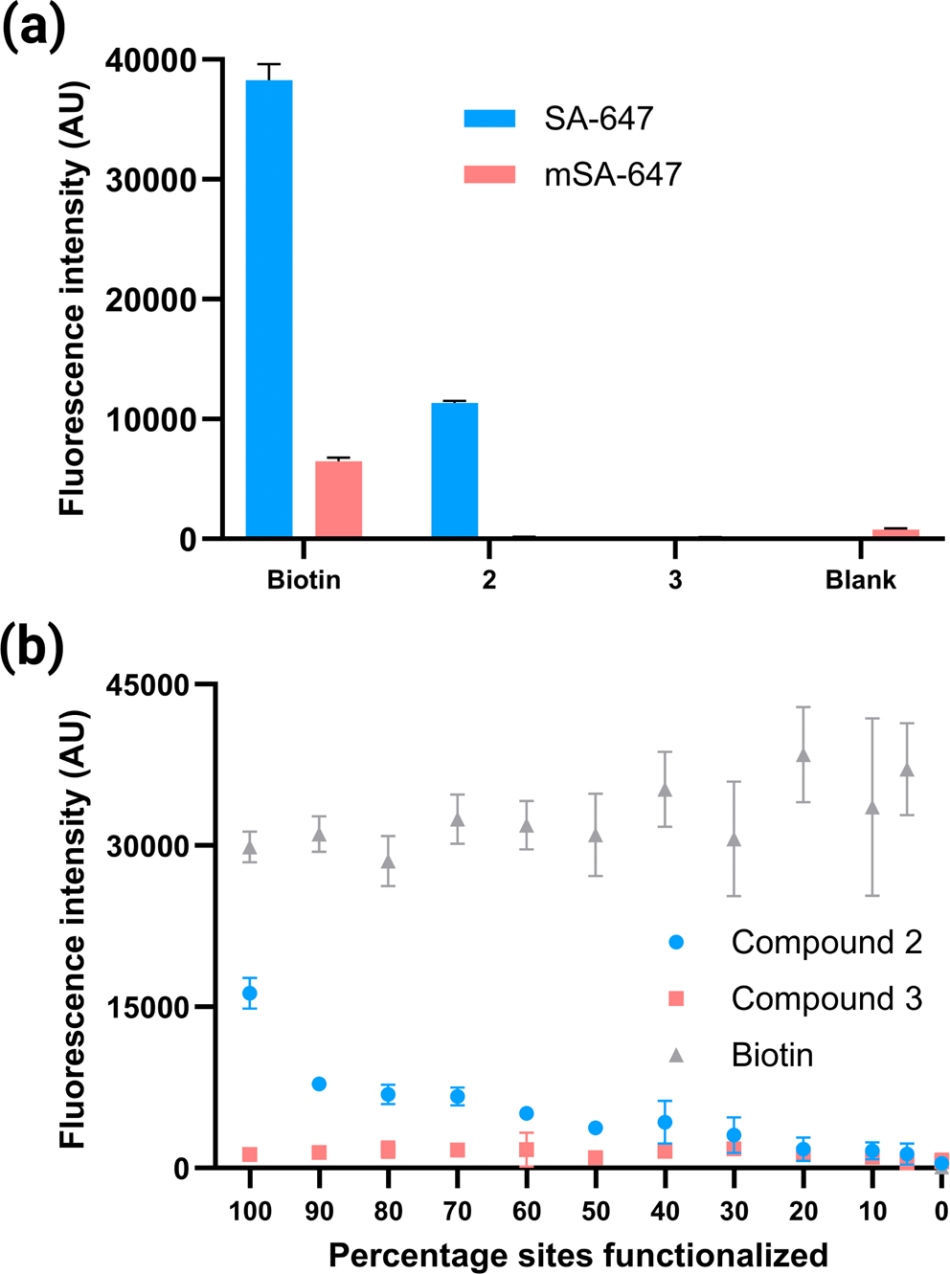
Stable binding of tetrameric SA to beads displaying compound **2** is highly dependent on avidity. (a) Binding of bead-displayed ligands to tetrameric (SA-647) and monomeric streptavidin (mSA-647). Fluorescence intensity of bead-displayed ligands was recorded after incubation with protein (500 nM) for 1 h in PBST at room temperature. Each compound was represented by 1 million 10 μm TentaGel beads, with 250,000 beads used per technical replicate. (b) Effect of ligand functionalization density on compound binding. Beads were functionalized with compound **2**, compound **3**, or biotin at varying surface densities (100% to 5%) and incubated with SA-647 (500 nM) for 1 h in PBST at room temperature as previously, before recording the fluorescence intensity.

To probe the importance of avidity in another way, we examined the binding of tetrameric SA-647 to beads displaying compound **2**, compound **3** or biotin, at different densities. “Dilution” of the ligand on the bead surface was achieved by using different amounts of the activated ester to modify the amino groups on the resin, achieving a gradient of site functionalization, ranging from 100% to 5%. As expected, retention of SA-647 by biotin was insensitive to ligand density (Figure 3b). In contrast, retention of SA-647 by compound **2** was strongly density-dependent (Figure 3b). The level of retained SA-647 was almost undetectable above background when **2** was linked to only 30% of the sites on the beads. These data strongly support an avidity-dependent mechanism for the retention of the protein by the low affinity ligand **2**.

### Distinguishing between High and Low Affinity Binding Events on Resin

We next asked if the bead binding assay could distinguish complexes with different affinities for SA. 10 μm TentaGel beads displaying compound **2**, compound **3**, the oxazole analogue of compound **2** (Figure S4), or the SA-binding macrocycle **1** shown in Figure 2a were exposed to different concentrations of SA-647 ranging from 0.1 to 1000 nM. After washing, the intensity of fluorescence retained on the beads was measured using a flow cytometer, and the results were plotted as the mean fluorescence intensity (MFI) on the beads vs the protein concentration. As seen in Figure 4a, dose-dependent binding of the protein was observed for beads displaying compounds **1**, **2**, and the oxazole analogue of **2**, but not for compound **3**. Macrocycle **1**, which binds SA with a *K*_D_ of about 600 nM in solution (more than 1000 times more tightly than compound **2**), binds SA-647 on resin at far lower concentrations than does compound **2**.

**Figure 4:**
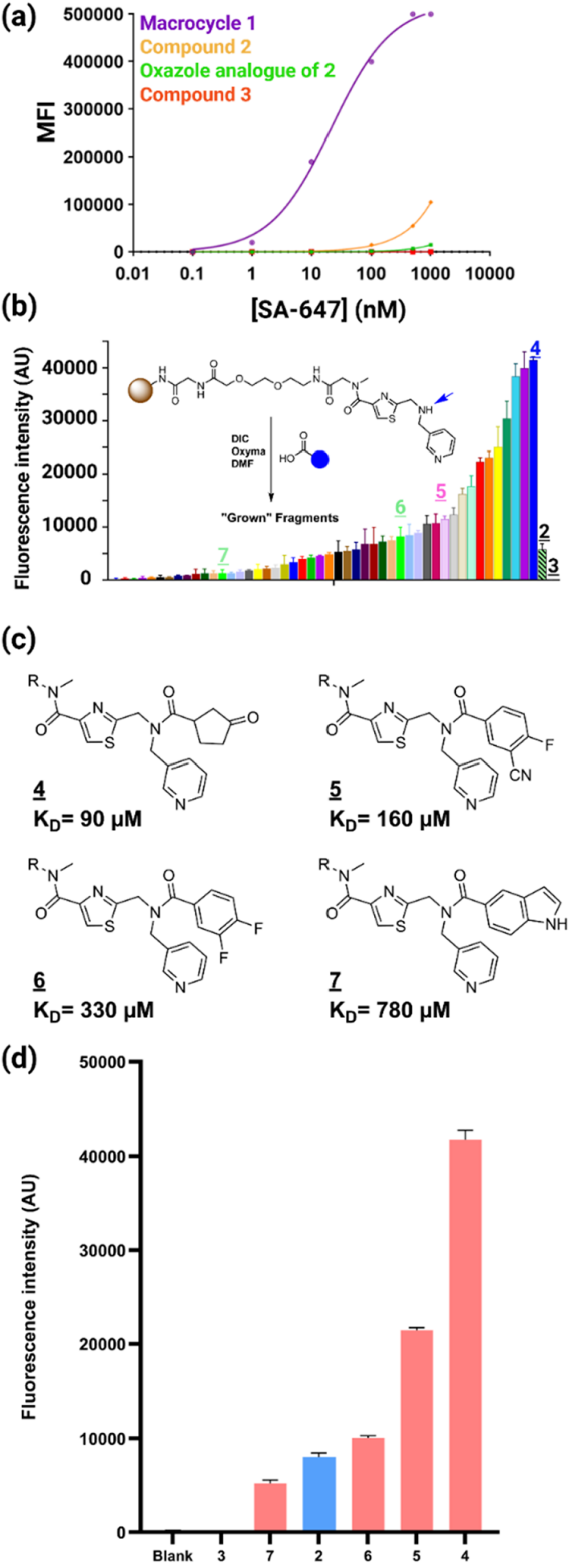
Retention of SA-647 by ligand-displaying TentaGel beads roughly correlates with the intrinsic *K*_D_ of the complex. (a) Dose-dependent binding of SA-647 to bead displayed macrocycle **1**, compound **2**, oxazole analogue of compound **2**, and compound **3** by FACS. (b) Fluorescence intensity of beads displaying grown versions of compound **2** after incubation with SA-647 (500 nM) for 1 h in PBST at room temperature. (c) Structures and the SPR *K*_D_ measurements of selected grown versions of compound **2**. (d) Fluorescence intensity of bead-displayed compounds **4**, **5**, **6**, and **7** along with controls.

However, compound **2** clearly retains SA-647 at lower protein concentrations than the oxazole analogue, which was derived from the minor hit in the macrocyclic DEL screen. This is a very weak ligand for SA with a *K*_D_ that is too high to measure by SPR (not shown). These data show that the bead binding assay can be used in at least a semiquantitative fashion to distinguish ligands with different affinities for the soluble protein.

### Bead-Binding Assays Allow the Discovery of Improved Ligands from “Expanded Ligand” Libraries

One of the most common methods for maturing a low affinity fragment into a lead compound is “fragment growing”,^4,36^ where additional units are added onto the original fragment to more fully fill the binding pocket and thus increase affinity. Given the results shown in Figure 4a, which demonstrate the bead-binding assay can clearly distinguish higher and lower affinity complexes at nonsaturating levels of soluble protein, we hypothesized that this type of screen could be supported by this platform.

To explore this idea, we substituted the acetic anhydride used in the final acylation step of compound **2** synthesis with 48 commercially available carboxylic acids (Figure 4b; the acids employed are shown in Figure S5). These compounds were made by parallel solid-phase synthesis in a 96-well microtiter plate format. Each type of bead was then exposed to SA-647 (100 nM) and, after washing, the degree of SA-647 binding was measured using a plate reader. The results are shown in Figure 4b.

Some of the “grown compounds” retained more SA-647, and some less, than the parent compound **2**. To determine if these intensities correspond to either an increase or decrease in intrinsic affinity for SA, a few of the grown compounds were synthesized in soluble form, and their binding to immobilized SA was measured by SPR. As shown in Figure 4c (see Supporting Figures S6–S9 for the raw SPR data), there was a good correlation between the fluorescence intensity of the resin and the binding affinity relative to the parent compound **2**. For example, grown compound **4**, which retained the most SA-647 in this experiment, was found by SPR to bind immobilized SA with a *K*_D_ of 90 μM, a 7.8-fold improvement over the parent compound **2**. In contrast, grown compound **7** retained less SA-647 than compound **2** when displayed on beads, and its intrinsic affinity for SA was found to be 780 μM by SPR. Bead-displayed compounds **6** captured less SA-647 than bead-displayed **4** but more than bead-displayed **2**. Its intrinsic *K*_D_ was found by SPR to be 330 μM, a value between that of the **2**·SA and **4**·SA complexes. We conclude from these data that the intensity of the fluorescent signal resulting from captured target protein is at least semiquantitatively related to the intrinsic affinity of the ligand for the protein in solution, and this assay will be useful for the discovery of improved ligands from fragment growth libraries when applied in a fragment screening assay.

### Discovery of Fragments That Bind a Homodimeric Protein

The experiments described above validate the idea that low affinity interactions between a bead-displayed ligand and a tetrameric protein can be detected easily even when the intrinsic *K*_D_ is in the high μM range. However, most proteins are not homotetramers or higher order homooligomers, raising the concern that this approach might be of limited target scope.

Many recombinant proteins are expressed and purified as fusions with Glutathione-S-Transferase (GST), which allows the facile purification of the protein on glutathione-agarose resin. GST is a native homodimer.^37^ Thus, it is of interest to ask if this technology can be applied to the discovery of low affinity ligands for dimeric proteins.

To explore this issue, we expressed and purified a N-terminal FLAG-tagged protein in which the human Rpn13 PRU (pleckstrin-like receptor for ubiquitin) domain is linked to the C-terminus of GST. Rpn13 is one of the Ubiquitin receptors of the 26S proteasome^38^ and has been the focus of significant interest as a potential oncology target.^39–41^ The N-terminal PRU domain of Rpn13 binds Ubiquitin and anchors the protein to the proteasome via interaction with a C-terminal domain in Rpn2.^42–44^ The C-terminal domain of Rpn13 binds and activates the Uch37 deubiquitylase. A few Rpn13 ligands have been reported, but none of these exhibit a *K*_D_ < 1 μM.^45–47^ Existing irreversible covalent ligands^48^ exhibit poor selectivity.^49^

A small panel of 94 bead-displayed fragments was created by coupling the activated esters of diverse carboxylic acids (Figure S10) to TentaGel beads using parallel solid-phase synthesis in a 96 well microtiter filter plate. The beads (one compound per well) were then incubated with GST-Rpn13 PRU fusion protein (500 nM) and, after washing, the amount of protein retained on the beads was measured after staining with Alexafluor-647 (A647)-labeled anti-FLAG antibody using a plate reader (Figure 5a). Two of the compounds, A8 and A88, exhibited markedly high fluorescence intensities that were more than three standard deviations (SDs) above the average signal in all of the wells. Three additional compounds (A63, A85, and A93) exhibited above-background fluorescence, though they did not exceed the “3 SD” threshold (Figure 5a). The individual fluorescence intensities of these screening hits, benchmarked against a bead-displayed peptide derived from Rpn2 that binds Rpn13 with nanomolar affinity,^42–44^ are shown in Figure 5b. The association of these bead-displayed fragments with FLAG-GST and human IgG (in this case binding was visualized using a labeled anti-IgG antibody) was also assessed to evaluate selectivity. As shown in Figure 5c, these signals were much lower than in the experiment employing GST-Rpn13 PRU, though fragments A8 and A88 did bind above-background levels of GST. Next a competition experiment was performed in which the GST-Rpn13 PRU fusion protein was preincubated with excess glutathione (1 mM) to determine if the fragments, to some extent, engage the peptide-binding site of GST. The results are shown in Figure 5d. Glutathione pretreatment significantly lowered the binding of fragments A8 and A88 to GST-Rpn13 fusion, suggesting a complex binding mode in which association of the fragment with the glutathione-binding site contributes to retention of the protein on the resin. We speculate that A8 and A88 probably bind to both GST and Rpn13, explaining the high signal in the original screen (Figure 5b). Finally, all five hit-displaying beads were incubated with only Alexa Fluor 647–labeled anti-FLAG antibody to confirm that the hits are not antibody binders (Figure S11).

**Figure 5:**
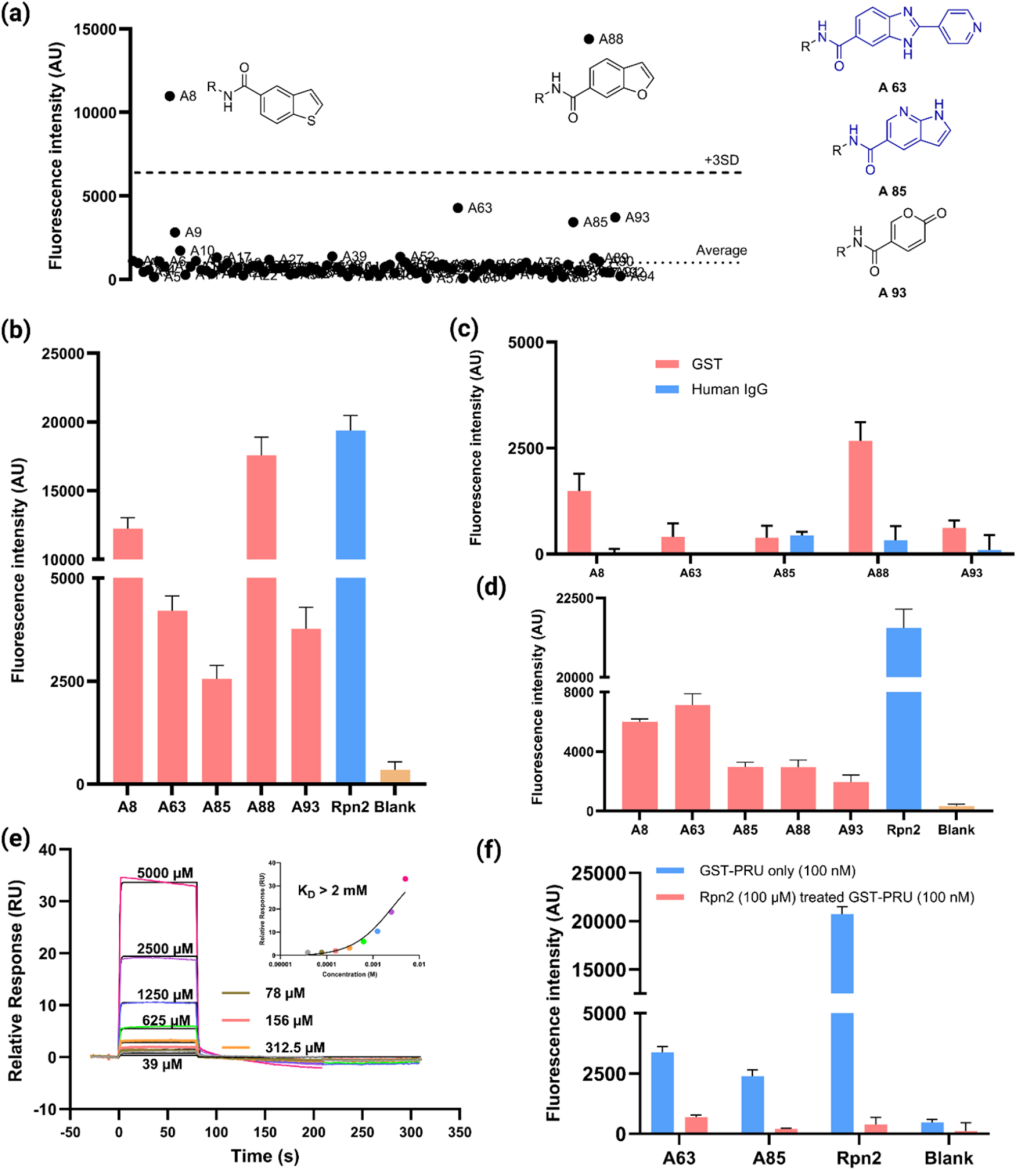
Weak-binding fragments can be effectively captured by screening with dimeric proteins. (a) Scatter plot showing the screening results of 94 bead-displayed fragments against the GST–Rpn13 PRU fusion protein, along with the structures of the top binders. (b) Fluorescence intensity of the identified bead-displayed weak binders and Rpn2-displayed beads after incubation with GST–Rpn13 PRU fusion protein (500 nM) for 1 h in PBST at room temperature. (c) Off-target binding of the identified weak binders; two dimeric off-targets (GST and human IgG) were screened under the same conditions as the target protein. (d) Competition experiment of bead-displayed identified weak binders using GST-Rpn13 fusion protein pretreated with glutathione (1 mM). (e) Monovalent affinity measurements for compound A85; SPR sensorgrams of A85 binding to immobilized Rpn13 PRU (inset shows the affinity fitting). (f) Competition experiment with A63-, A85-, and Rpn2-displayed beads using GST–Rpn13 PRU fusion protein pretreated with Rpn2 (100 μM).

While these results do not rule out the potential utility of A8 and A88 as a starting point for Rpn13 ligand development, we chose to focus on A63 and A85 for further analysis. A93 was discarded because we were concerned that this might be a covalent ligand.^50^ As shown in Figure 5e, SPR analysis of soluble A85 binding biotinylated Rpn13 PRU immobilized on a SA chip weakly, with a *K*_D_ of >2 mM. A similar result was obtained with A63 (Figure S12). This result validates binding of the small molecules to Rpn13 PRU that is not fused to GST. Most importantly, for the purposes of this study, these data validate that the bead-display system is capable of detecting even quite weak fragment-protein interactions when the protein is a homodimer.

As mentioned above, the Rpn13 PRU domain binds to the proteasome via association with a C-terminal peptide tail from Rpn2.^44^ To determine if A63 and A85 recognize this binding surface, a competition experiment was carried out in which bead-displayed A63, A85, or (as a control) Rpn2 peptide were treated with GST–Rpn13 PRU that had, or had not, been preincubated with excess soluble Rpn2 peptide (100 μM). The amount of protein retained on the resin was then evaluated using the same protocol described above. As shown in Figure 5f, the presence of the soluble peptide competitor strongly suppressed binding to all of the ligands tested. These results suggest that both A63 and A85 recognize the Rpn2-binding surface of Rpn13.

### High-Throughput Screening of Fragments against SA via FACS

While the one compound per well screening format employed for the Figures 4 and 5 experiments works well, it will limit throughput for the analysis of much larger fragment libraries in the future. Thus, we next sought to develop a screening workflow that would allow many different bead-displayed fragments to be screened in a single tube. Toward this end, we synthesized a small library of 51 bead-displayed fragments (Figure S13). A photocleavable linker was placed between the fragment and the bead (Figure 6a). Beads displaying compounds **2**, **3**, and the oxazole analog of compound **2** were also constructed with the same architecture. Beads displaying all of these compounds were then mixed together in a single Mobicol tube (approximately 25,000 of each bead type).

**Figure 6:**
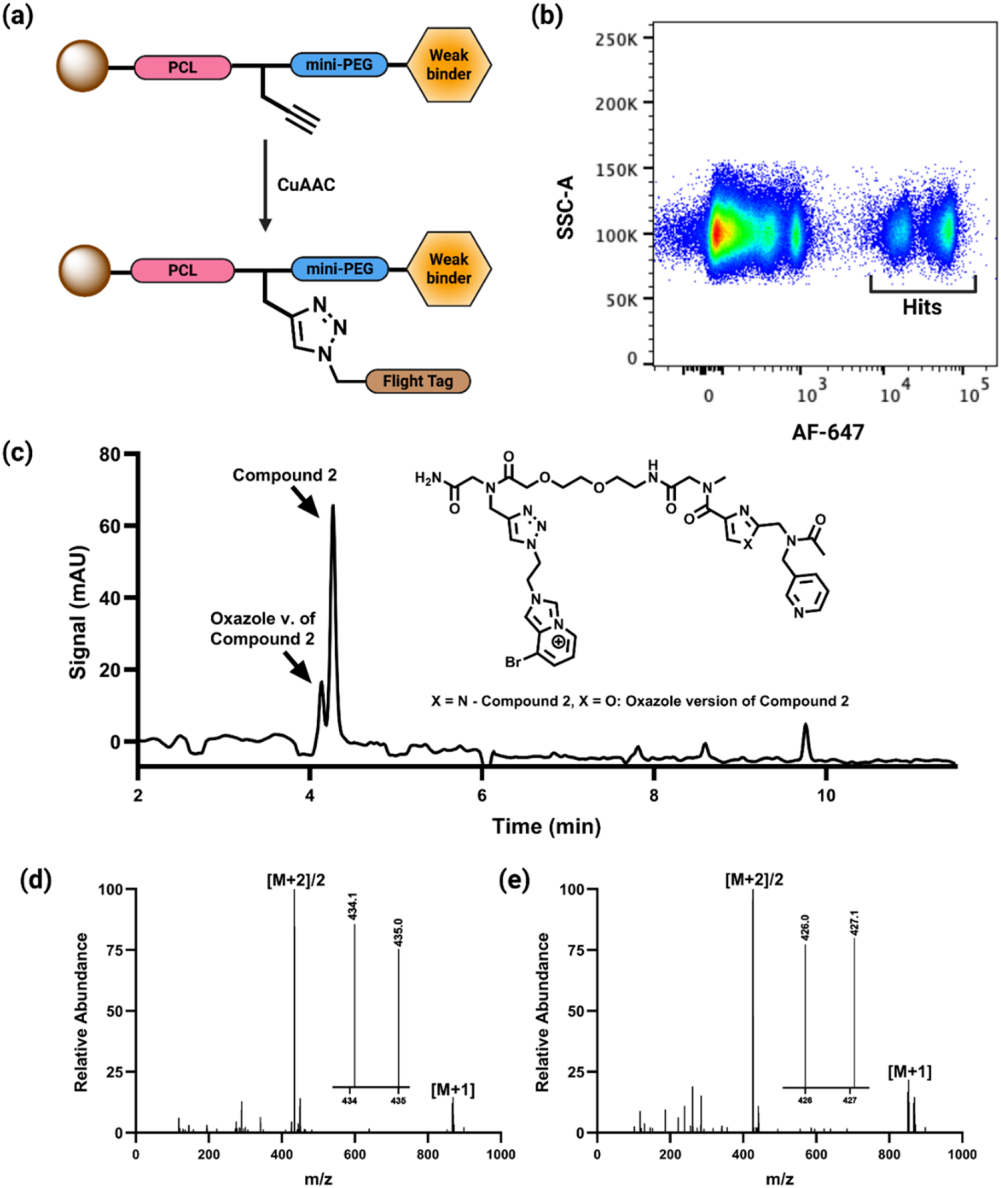
High-throughput screening (HTS) of pooled bead-displayed fragment size compound library via FACS and LC-MS hit identification. (a) Schematic representation of the architecture of the beads used for this experiment. Each compound was conjugated to TentaGel resin via a photocleavable linker (PCL), followed by a mini-PEG spacer and a clicked Flight Tag (added after sorting). (b) Flow cytometry dot plot (SSC-A vs AF647-A) showing the separation of SA-647-bound (high-fluorescence) and unbound bead populations. (c) LC trace of the photocleaved eluent from the sorted, SA-647-bound bead population, highlighting the presence of enriched ligand peaks. (d) The mass spectrum corresponds to the LC peak of the compound **2** derivative, with the inset showing the characteristic bromine isotopic distribution of the **M+2/2** signal. (e) The mass spectrum corresponds to the LC peak of the oxazole analogue of compound **2**, with the inset showing the characteristic isotopic the characteristic isotopic distribution of the **M+2/2** signal.

The resin was incubated with SA-647 (500 nM, 300 μL) for 1 h at room temperature with gentle rotation. Following thorough washing to remove unbound protein, the pooled beads were passed through a fluorescence-activated cell sorter (FACS), gated to collect those that displayed a high level of red fluorescence (labeled “Hits” in Figure 6b). This FACS-based hit recovery protocol has been used previously in one bead one compound library screening.^29,34,51^ As can be seen in Figure 6b, there were two distinct populations of red fluorescent beads, but these were collected as a single batch.

Prior to releasing the compounds from the resin for analysis, we “clicked” a brominated imidazopyridinium unit (IP^+^)^52^ to the alkyne unit in the linker of these beads via copper-catalyzed azide–alkyne cycloaddition (Figure 6a,c insert).^53^ This was done for three reasons. The first was to add a chromophore to molecules so that, after release from the beads, they could be detected sensitively using a UV detector on the LC trace. The second was to boost the ionization efficiency of these molecules by virtue of adding the positive charge. Third, the characteristic isotope ratio of bromine unequivocally identified compounds in the mass spectrometer as being library components. Peaks that do not exhibit the characteristic “bromine doublet” are noise and can be ignored.

After attachment of the IP^+^ tag, the beads were washed with a 1:1 mixture of DMF and DCM and irradiated at 365 nm (4.5 × 10^3^ μJ cm^–2^) for 1 h in a 3:1 DCM/MeOH mixture to release the ligands from the beads. After evaporation of solvents, the eluate was redissolved in water and analyzed by LC-MS.

As shown in Figure 6c, two significant peaks were observed in the chromatogram. Their masses revealed them to be the IP^+^-modified derivatives of compound **2** (major peak) and the oxazole analogue of **2** (minor peak). Their molecular ions displayed the distinctive isotope split of a bromine-containing compound (Figure 6d,e). None of the other, more minor peaks in the LC displayed this pattern (not shown), meaning they are not compounds in the library and represent some sort of impurity. None of the other compounds in the library were identified in the collected hit pool. These data demonstrate that these low affinity SA-binding compounds can be recovered from a ≈50-plex screen carried out in a single tube.

## Discussion

Fragment-based drug development is an appealing workflow for the development of new drugs and probe molecules. Many methods for the analysis of higher affinity small molecule-protein interactions cannot detect these low affinity, kinetically unstable complexes. Indeed, almost all effective methods for fragment discovery rely on biophysical techniques capable of analyzing binding under equilibrium conditions, such as NMR or SPR. While highly effective, screening thousands of candidate fragments using these techniques requires relatively expensive, specialized infrastructure that is not available to many investigators. Therefore, there remains a need for new approaches to screening fragment collections for ligands to a protein of interest (POI).

Here, we demonstrate that a simple pull-down assay, using TentaGel-displayed small molecules and fluorescently labeled soluble proteins, is capable of detecting low affinity binding events (*K*_D_s in the high μM to low mM region) typical of fragment-protein complexes, so long as the POI is a multimer. Using fluorescently labeled tetrameric SA as a model target, we readily detected binding of the protein to a TentaGel bead-displayed compound, **2**, whose intrinsic affinity for SA is quite weak (*K*_D_ of 706 μM), with an excellent signal-to-noise ratio relative to the nonbinding control compound **3**. Binding of bead-displayed **2** to SA was detectable even at streptavidin concentrations 14,000-fold below the intrinsic *K*_D_ of the complex and the association was long-lived, with a half-life far in excess of 24 h (Figure 2).

The mechanistic basis for this enhanced sensitivity appears to be firmly rooted in avidity effects, as evidenced by two key observations. First, binding to resin-displayed compound **2** was not observed when monomeric streptavidin was substituted for the tetramer (Figure 3a). Second, the apparent affinity of SA-647 for beads displaying compound **2** was highly dependent on the number of ligand molecules attached to the resin, whereas this was not the case for beads displaying the intrinsically high-affinity SA ligand biotin (Figure 3b).

A common strategy to mature fragment hits into higher-affinity leads is to produce a collection of derivatives of the fragment hit containing potential additional binding elements and screen them for improved ligands. We were gratified to find that this platform supports this workflow and is able to differentiate between higher and lower affinity ligands when the bead-binding experiment is done at a subsaturating protein concentration (Figure 4). Indeed, comparison of the amount of SA-647 captured on a bead by derivatives of compound **2** with their intrinsic solution *K*_D_s for SA showed a rough correlation between the two values (Figure 4). Thus, this platform can support the first two steps of a typical FBDD campaign, fragment discovery and growth.

Since most interesting drug targets are not homotetramers or higher order multimers (though some are),^54–57^ a concern is the target scope of this methodology. Thus, we evaluated it is efficacy in a screen of a small fragment library against a GST fusion of a so far “undruggable” target, the PRU domain of Rpn13. GST is a native homodimer and we hoped that this would provide a sufficiently large avidity effect to allow detection of low affinity small molecule-protein complexes. This was indeed the case. A screen of a small library of carboxylic acid-derived fragments attached to TentaGel beads resulted in the discovery of Rpn13-PRU binding fragments. SPR analysis of two of these fragments demonstrated they bound Rpn13-PRU with a *K*_D_ in excess of 2 mM (Figures 5 and S12) demonstrating the high level of sensitivity of the assay using even a dimeric target. This finding suggests that most monomeric proteins or protein domains will be viable targets using this methodology since GST fusion proteins are readily expressed in *Escherichia coli* and easily purified by glutathione affinity chromatography. In the future, we will include saturating levels of glutathione in the buffer employed for the fragment screens to block association of the bead-displayed compounds with the peptide-binding site of GST. This clearly contributed to the signals we saw for A8 and A88 binding to GST-Rpn13 PRU (Figure 5).

When carried out in a plate-based, one compound per well format with binding monitored using a plate reader, it would be straightforward to screen a few thousand compounds, a throughput comparable to that achievable using state-of-the-art SPR, but using much less expensive instrumentation. Nonetheless, throughput would be improved and the cost of screening reduced if this could be done in batch mode, mixing dozens to hundreds of different compound-displaying beads in a single tube. The data in Figure 6 demonstrate the feasibility of this approach. Beads displaying ≈50 different compounds, including **2**, the oxazole analogue of **2** and the negative control **3**, in a single tube were incubated with labeled SA and beads that retained the labeled protein were separated from those that did not by FACS. After addition of an IP^+^-based chromophore/flight tag, and release from the resin, the SA-binding compounds were readily identified by LC-MS. Since FACS-based methods have been employed to screen DNA-encoded one bead one compound libraries,^29,34,58^ these data argue that it will be possible to screen many thousands of fragments in a single tube if they are DNA-encoded, substituting deep sequencing of the encoding tags for LC-MS analysis of the hit pool.

Of course, it is also important to point out the limitations of the current version of this platform. The most obvious is that a large collection of diverse, TentaGel-displayed fragments does not currently exist and will have to be constructed. This is a barrier to entry that does not exist for some established methods that employ unmodified fragments that can simply be purchased. We hope that this disadvantage will be rectified in the near future through commercial offerings of TentaGel-displayed fragment libraries. We are in the process of creating a large collection of structurally diverse bead-displayed fragments and will report these efforts in due course.

Another limitation, shared by many other fragment discovery methods, is that our method does not provide structural information, as is the case for SAR by NMR. Such information is invaluable in planning fragment growth and/or linkage campaigns downstream of discovery of the initial fragment, which is one of the reasons SAR by NMR is such a powerful workflow for FBDD.^17,36,59^ However, we suggest that it may be more resource- and time-efficient to carry out the initial fragment discovery work using this method, then transition to structural elucidation of the hit fragment-protein complexes.

In conclusion, we have shown that simple pull-down assays can be used to identify low affinity, low molecular weight ligands for multimeric proteins, including dimeric GST fusion proteins. The methodology employs equipment that is available in most chemistry and biochemistry laboratories and requires only small amounts of protein. We anticipate that, once large libraries of TentaGel-displayed fragments become available, this technology will contribute significantly to the facile discovery of protein-binding fragments as starting points for drug development.

## Experimental Section

### General

Unless otherwise noted, all starting materials, chemical/bio reagents, and solvents were purchased from commercial sources and used without further purification. TentaGel beads were purchased from Rapp Polymere GmbH (Germany). Disposable reaction columns (TORVIQ and ISOLUTE) were used as reaction vessels for solid-phase synthesis.

NMR spectra for characterization were measured in DMSO-*d*_6_ solutions using a Bruker AVANCE NEO 600 MHz instrument with TCI Cryoprobe and a Bruker AVANCE NEO 400 MHz instrument.

An Agilent 1100 series HPLC equipped with Agilent ZORBAX SB-C18 column (Part number: 861953–902, 4.6 mm × 100 mm, 3.5 μm) and an electrospray ionization (ESI) source (Agilent Technologies 6120 Quadrupole) was used to obtain mass spectra for small molecules. The purity of the samples was tested in the LC-MS instrument using a solvent gradient of 100% water (0.1% formic acid) and 100% acetonitrile (0.1% formic acid) (100% water to 100% acetonitrile over 10 min). All compounds are >95% pure by LC-MS analysis.

Small molecule column chromatography (Prep-HPLC) was conducted using a Waters HPLC system equipped with a Waters 1525 binary HPLC pump and a 2487 dual λ absorbance detector, or a 2998 photodiode array detector. Water (with 0.1% Trifluoroacetic acid) and acetonitrile (with 0.1% Trifluoroacetic acid) were used as solvents A and B, respectively.

Fluorescence intensity measurements were recorded in a Tecan Spark multiwell plate reader equipped with appropriate excitation and emission filters (excitation at 650 nm, emission at 665 nm). FACS experiments were conducted using a BD FACS Fusion cell sorter.

### SPR Measurements

#### Streptavidin

The SPR binding affinity testing was conducted using the BIAcore 1K system (Cytiva) with BIAcore Insight Control Software. Streptavidin (50 μg/mL in 10 mM pH 5.0 NaOAc) was immobilized on a CM5 sensor chip (Cytiva) via amide coupling, using HBS-EP+ (10 mM HEPES, 150 mM NaCl, 3 mM EDTA, and 0.05% v/v Surfactant P20) as the immobilization buffer. Approximately 1000 RU of immobilization was achieved. Testing compounds were evaluated using the multiple-cycle kinetic/affinity method in PBS-P+ running buffer (20 mM phosphate buffer, 2.7 mM KCl, 137 mM NaCl, and 0.05% Surfactant P20), with a 200 s association time, 200 s dissociation time, and a flow rate of 45 μL/min. Binding affinities for the testing compounds were determined by fitting the sensor grams to both the 1:1 binding kinetics and steady-state affinity models provided in the BIAcore Insight Evaluation Software.

#### Rpn13-PRU

4000–5000 Ru of Biotinylated Rpn13-PRU (50 μg/mL in running buffer (20 mM phosphate buffer with 2.7 mM KCl, 137 mM NaCl, 0.05% Surfactant P20, 1 mM DTT and 1 mM EDTA)) was captured on a SA sensor chip (Cytiva). Testing compounds were evaluated using the multiple-cycle kinetic/affinity method in running buffer with indicated association and dissociation time, and a flow rate of 40 μL/min. Data was analyzed with the BIAcore Insight Evaluation Software and the binding affinities were obtained by fitting the sensor grams to the 1:1 binding kinetics, steady-state affinity models or steady-state affinity model with constant *R*_max_.

### General Solid-Phase Synthesis Procedures

#### Fmoc Deprotection

The Fmoc group was deprotected by 20% piperidine in DMF at room temperature (2 × 10 min). The beads were extensively washed by DCM (2×) and DMF (2×).

#### Acylation of Deprotected Amines

Acid building blocks (10 equiv to resin loading), *N*,*N*′-Diisopropylcarbodiimide (DIC, 10 equiv), and Ethyl (hydroxyimino)­cyanoacetate (Oxyma, 10 equiv) were mixed in DMF for 5 min. Then, the resulting solution was added to the beads and mixed. The resulting suspension was shaken for 1 h at 37 °C. Upon reaction completion, the resin was washed with DCM (2×) and DMF (2×).

#### Halide Displacement

Primary amine building blocks (500 mM in DMF) with DIPEA (2% in DMF) were added to the bead-displayed chloromethyl starting materials (Ex: step 5 of Scheme 1), and the resulting suspension was shaken for 3 h at 37 °C. Upon reaction completion, the resin was washed with DCM (2×) and DMF (2×).

#### Capping with Acetic Anhydride

A mixture of acetic anhydride (10% in DMF) and DIPEA (2% in DMF) was added to the bead-displayed amine (Ex: final step in Scheme 1), and the resulting suspension was shaken for 30 min at room temperature. Upon reaction completion, the resin was washed with DCM (2×) and DMF (2×).

### Synthesis of Compound **2** on 10 μm Beads

Bead-displayed compound **2** was synthesized using general solid-phase synthesis procedures and outlined in SI Scheme 1.

### Synthesis of Compound **3** in 10 μm Beads

Bead-displayed compound 3 was synthesized using general solid-phase synthesis procedures and outlined in SI Scheme 2.

### Synthesis of the Oxazole Version of Compound **2** in 10 μm Beads

Bead-displayed oxazole version of compound 2 was synthesized using general solid-phase synthesis procedures and outlined in SI Scheme 3.

### On-Bead Compound Extension (Outlined in SI Scheme 4)

Bead-displayed compound **8** was synthesized as described in SI Scheme 1. Beads (1 mg) displaying compound **8** were added to each well of a preactivated (with DCM) hydrophobic multiwell synthetic plate (Millipore Sigma MSRPN04). The beads were treated with a premixed solution of the acid building block (10 equiv relative to resin loading), *N*,*N*′-diisopropylcarbodiimide (DIC, 10 equiv), and ethyl (hydroxyimino)­cyanoacetate (Oxyma, 10 equiv) in DMF (150 μL). The suspension was shaken for 1 h at 37 °C. After draining the reaction mixture, the beads were treated with a fresh premixed solution of the acid building block, DIC, and Oxyma in DMF under the same conditions (1 h at 37 °C) to ensure complete acylation of the secondary amine. Upon completion, the resin was washed with DCM (2×) followed by DMF (2×).

### On-Bead Fragment Library Synthesis for GST-Rpn13 PRU Fusion Screening

All fragment library compounds were synthesized on the bead-displayed mini-PEG spacer. Beads (1 mg) displaying the mini-PEG spacer were added to each well of a preactivated (with DCM) hydrophobic multiwell synthetic plate (Millipore Sigma MSRPN04). The beads were treated with a premixed solution of the acid building block (10 equiv relative to resin loading), *N*,*N*′-diisopropylcarbodiimide (DIC, 10 equiv), and ethyl (hydroxyimino)­cyanoacetate (Oxyma, 10 equiv) in DMF (150 μL). The suspension was shaken for 1 h at 37 °C. After draining the reaction mixture, the beads were treated with a fresh premixed solution of the acid building block, DIC, and Oxyma in DMF under the same conditions (1 h at 37 °C) to ensure complete acylation of the amine. Upon completion, the resin was washed with DCM (2×) followed by DMF (2×).

### On-Bead Photocleavable Mock Library Synthesis

All mock library compounds, including the control compounds (compound 2, compound 3, the oxazole analog of compound 2, and a blank), were synthesized on the bead-displayed spacer compound **9**, as shown in SI Scheme 5. Bead-displayed compound **9** was synthesized using a five-step solid-phase synthetic route, following the general procedure, with Fmoc-Photo-Linker (CAS# 162827–98–7) used in step 2. Control compounds were synthesized on 10 mg of compound-9-displaying 10 μm TentaGel beads in separate tubes. Acylation reactions with commercial carboxylic acids were performed in a multiwell plate as described previously.

### Synthesis of Mini-PEG Attached Compound **2**

Compound 2 with the mini-PEG spacer was synthesized on 250 mg of Rink amide AM resin (CHEM IMPLEX, Cat# 06761; 0.7–1.0 mequiv/g, 100–200 mesh, 0.722 mmol/g loading capacity) following general solid-phase procedures. After completion of the synthesis, the compound was cleaved from the resin using a cocktail of TFA (95%), TIPS (2.5%), and water (2.5%) (v/v) for 45 min at room temperature. TFA was removed under a stream of argon, and the crude product was precipitated by trituration with diethyl ether. After removal of ether, the residue was dissolved in water and purified by preparative HPLC using a linear gradient of acetonitrile/water (5:95 to 60:40) over 30 min. Product-containing fractions were lyophilized to afford compound 2 (83 mg, 82%) as a straw-colored semisolid in TFA salt form. ^1^H NMR (400 MHz, DMSO-*d*_6_, 100 °C) δ 8.72–8.52 (m, 2H), 7.98 (s, 2H), 7.74–7.45 (m, 3H), 6.89 (s, 2H), 5.02–4.64 (m, 4H), 4.17 (s, 2H), 3.93 (s, 2H), 3.74 (d, *J* = 5.4 Hz, 2H), 3.67–3.55 (m, 4H), 3.49 (t, *J* = 5.9 Hz, 2H), 3.29 (q, *J* = 5.7 Hz, 2H), 3.05 (s, 3H), 2.17 (s, 3H); HRMS (Q Exactive Orbitrap) *m*/*z*: [M + H]^+^ calculated for C_24_H_34_N_7_O_7_S 564.22404; found 564.22228; *t*_R_ = 4.1 min.

The room temperature NMR spectra (both ^1^H and ^13^C) of these compounds show complex behavior due to the presence of multiple conformers in solution and slow rotation/exchange of those conformers. However, upon acquiring ^1^H NMR at elevated temperatures, the spectra became rather simple, yet not all compounds were stable at elevated temperatures. Therefore, compound characterization was primarily conducted by LC-MS. As a representative example, only the NMR analysis of the mini-PEG attached compound 2 is reported in SI Figures 15, 16, and 17.

### Synthesis of Mini-PEG Attached Compound **3**

Compound 3 with the mini-PEG spacer was synthesized following the methods stated in the synthesis of mini-PEG attached compound 2 to afford fragment 3 (88 mg, 87%) as a clear oil. LC-MS (ESI) *m*/*z*: [M + H]^+^ calculated for C_24_H_35_N_6_O_7_S 563.2; found 563.2; *t*_R_ = 5.9 min

### Synthesis of Mini-PEG Attached Compound **4**

Compound 4 with the mini-PEG spacer was synthesized following the methods stated in the synthesis of mini-PEG attached compound 2 to afford compound 4 (90 mg, 79%) as a brown solid. HRMS (Q Exactive Orbitrap) *m*/*z*: [M + H]^+^ calculated for C_28_H_38_N_7_O_8_S 632.25026; found 632.24850; *t*_R_ = 4.3 min

### Synthesis of Mini-PEG Attached Compound **5**

Compound 5 with the mini-PEG spacer was synthesized following the methods stated in the synthesis of mini-PEG attached compound 2 to afford compound 5 (70 mg, 58%) as an orange-colored thick oil. LC-MS (ESI) *m*/*z*: [M + H]^+^ calculated for C_30_H_34_FN_8_O_7_S 669.2; found 669.2; *t*_R_ = 5.2 min.

### Synthesis of Mini-PEG Attached Compound **6**

Compound 6 with the mini-PEG spacer was synthesized following the methods stated in the synthesis of mini-PEG attached compound 2 to afford compound 6 (92.5 mg, 77%) as a yellow-colored thick oil. LC-MS (ESI) *m*/*z*: [M + H]^+^ calculated for C_30_H_34_FN_8_O_7_S 662.2; found 662.2; *t*_R_ = 5.3 min.

### Synthesis of Mini-PEG Attached Compound **7**

Compound 7 with the mini-PEG spacer was synthesized following the methods stated in the synthesis of mini-PEG attached compound 2 to afford compound 7 (68.3 mg, 56%) as a white solid. HRMS (Q Exactive Orbitrap) *m*/*z*: [M + H]^+^ calculated for C_30_H_34_FN_8_O_7_S 665.22552; found 665.24887; *t*_R_ = 4.9 min

### Synthesis of Mini-PEG Attached Compound **A63**

Compound A63 with the mini-PEG spacer was synthesized following general acylation methods stated above to afford compound A63 (47.9 mg, 53%) as a clear, thick oil. LC-MS (ESI) *m*/*z*: [M + H]^+^ calculated for C_23_H_28_N_7_O_6_ 498.2; found 498.1; *t*_R_ = 4.4 min.

### Synthesis of Mini-PEG Attached Compound **A85**

Compound A85 with the mini-PEG spacer was synthesized following the general acylation methods stated above to afford compound A85 (46.1 mg, 77%) as a thick pale-yellow oil. LC-MS (ESI) *m*/*z*: [M + H]^+^ calculated for C_18_H_24_N_6_O_6_ 421.2; found 421.1; *t*_R_ = 4.6 min.

### Synthesis of Rpn2 Peptide

Wilde-type human Rpn2 peptide^44^ was synthesized using 250 mg of Rink amide AM resin (CHEM IMPLEX, Cat# 06761; 0.7–1.0 mequiv/g, 100–200 mesh, 0.9 mmol/g loading capacity) following general solid-phase procedures. After completion of the synthesis, the compound was cleaved from the resin using a cocktail of TFA (95%), TIPS (2.5%), and water (2.5%) (v/v) for 45 min at room temperature. TFA was removed under a stream of argon, and the crude product was precipitated by trituration with diethyl ether. After removal of ether, the residue was dissolved in water and purified by preparative HPLC using a linear gradient of acetonitrile/water (5:95 to 70:40) over 30 min. Product-containing fractions were lyophilized to afford Rpn2 peptide (171.3 mg, 45%) as a white solid in TFA salt form. LC-MS (ESI) *m*/*z*: [M + 2]/2 calculated for C_78_H_108_N_16_O_26_ 843.4; found 843.3; *t*_R_ = 5.3 min.

### Solution Phase Synthesis

#### Synthesis of Flight-Tag for MS (Compound **10**)

2-Azidoethanamine hydrochloride (122.56 mg, 1 mmol) and 3-bromopyridine carbaldehyde (204.6 mg, 1.1 mmol) were dissolved in water (0.9 mL) and AcOH (0.1 mL) in a 20 mL glass vial. To this solution, formaldehyde (37% w/w in water, 0.1 mL, 2.5 mmol) and DIPEA (0.209 mL, 1.2 mmol) were added, and the resulting solution was stirred at 37 °C for 18 h. The resulting crude product was diluted with water and filtered. The crude filtrate was purified using preparative HPLC using a linear gradient of acetonitrile/water (5:95 to 60:40) over 30 min. Product-containing fractions were lyophilized to afford the target compound as TFA salt (367 mg, 96%) as a clear oil. ^1^H NMR (600 MHz, DMSO-*d*_6_) δ 9.95 (d, *J* = 1.7 Hz, 1H), 8.69 (d, *J* = 7.2 Hz, 1H), 8.50 (s, 1H), 7.66 (d, *J* = 7.3 Hz, 1H), 7.13 (t, *J* = 7.2 Hz, 1H), 4.72–4.66 (m, 2H), 4.05–3.97 (m, 2H) ^13^C­{^1^H} NMR (151 MHz, DMSO-*d*_6_) δ 129.23, 128.99, 127.48, 123.98, 117.66, 115.19, 110.10, 49.80, 49.62; HRMS (Q Exactive Orbitrap) *m*/*z*: [M]^+^ calculated for [C_9_H_9_BrN_5_]^+^ 266.00413; found 266.00397.

### Protein Expression and Purification

Recombinant GST-Rpn13 PRU protein was expressed in BL21 (DE3) codon plus RP cells using a pET3a expression vector cloned with GST-Rpn13 PRU bearing an N-terminal FLAG tag and a C-terminal His6 tag for purification (pET3a-FLAG-GST-Rpn13 PRU-His6).

Recombinant Rpn13 PRU protein was expressed in BL21 (DE3) codon plus RP cells using a pET3a expression vector cloned with Rpn13 PRU bearing an N-terminal FLAG tag and C-terminal His10 tag for purification and Avi tag for biotinylation (pET3a-FLAG-Rpn13 PRU-His10-Avi).

Recombinant GST protein was expressed in BL21 (DE3) codon plus RP cells using a pET3a expression vector cloned with GST bearing an N-terminal FLAG tag and C-terminal His6 tag for purification (pET3a-FLAG-GST-His6).

In summary, BL21­(DE3) CodonPlus-RP cells harboring expression vectors were grown overnight in LB medium (5 mL) supplemented with ampicillin (100 μg/mL) and chloramphenicol (25 μg/mL). The overnight culture was used to inoculate 1 L LB medium containing the same antibiotics, and cells were grown at 37 °C with shaking at 170 rpm until the OD_600_ reached ∼0.6. Protein expression was induced with IPTG (1 mM final concentration) and continued expression for 4 h at 24 °C. Cells were harvested by centrifugation (8000 rpm, 15 min, 4 °C), the pellet snap-frozen in liquid nitrogen, and stored at −80 °C until purification.

Frozen bacterial cell pellets were thawed on ice, resuspended, and homogenized in chilled lysis buffer [50 mM sodium phosphate, 300 mM NaCl, 1.5 mM imidazole, 1 mM PMSF, 10 mM β-mercaptoethanol, and 1 mg/mL lysozyme, pH 8.0]. The suspension was stirred vigorously with a magnetic stir bar for 30 min at 4 °C, followed by sonication on ice (three 1 min bursts at 40% power, with intermittent mixing and vortexing). The crude lysate was clarified by centrifugation at 9000 rpm for 30 min at 4 °C using a fixed-angle rotor.

Ni–NTA resin slurry (Qiagen, 2 mL) was equilibrated by washing once with 50 mL dd H_2_O and once with 50 mL lysis buffer. Resin was collected by centrifugation (200*g*, 3 min, 4 °C), and all steps were performed on ice unless otherwise stated. Clarified lysate was added to the equilibrated resin and incubated at 4 °C with gentle tumbling for 2 h. The resin was washed three times with 10 mL wash buffer (50 mM sodium phosphate, 300 mM NaCl, 10 mM β-mercaptoethanol, 20 mM imidazole, pH 8.0), and each wash fraction was collected separately. Bound protein was eluted sequentially with 5 mL wash buffer containing increasing imidazole concentrations: 100 mM (1 × 5 mL), 250 mM (1 × 5 mL), 500 mM (2 × 5 mL), and 1 M (1 × 5 mL).

Protein purity in elution fractions was determined by SDS-PAGE, and fractions of interest were pooled. Desalting was performed using PD-10 Sephadex G-25 columns (Cytiva), followed by concentration with Amicon Ultra centrifugal filters (10 kDa MWCO, Millipore). Purified protein was exchanged into HBS–TE buffer (10 mM HEPES–HCl, pH 7.4, 150 mM NaCl, 1 mM TCEP, 1 mM EDTA) and stored at −80 °C.

Protein quality control was assessed by fluorescence polarization using a fluorescein-conjugated Rpn2 peptide, as previously described.^44^

The FLAG–Rpn13 PRU–Avi–His10 construct was biotinylated using a commercially available enzymatic protein biotinylation kit (Millipore, CS0008) according to the manufacturer’s instructions.

### Screening

#### Screening Using Multiscreen Plates for Streptavidin

General notes:

- At each step, contents were mixed by pipetting up and down 3–5 times.
- All steps following the addition of SA-647 were conducted in the dark.
- SA-647 and mSA-647 were purchased from commercial sources; (SA-647: ThermoFisher cat#S3235, mSA-647: Millipore SAE178).

A 96-well Multiscreen hydrophilic assay plate (MilliporeSigma, MSBVN12) was activated by treating each well with 150 μL of 70% (v/v) ethanol in water. The solvent was removed under vacuum, and the wells were washed with PBST (3 × 150 μL). Fresh PBST (150 μL) was then added to each well.

Bead-displayed compounds (∼1 × 10^6^ beads in 100 μL DMF) were added individually to separate wells (one compound per well). The plate was drained and washed again with PBST (3 × 150 μL), followed by the addition of 150 μL PBST per well. Plates were shaken overnight at ambient temperature on an orbital plate shaker.

The next day, wells were drained and incubated with 150 μL of a 1:1 (v/v) solution of PBST:PBS blocking buffer (Thermo Scientific, 37538) for 1 h at room temperature. After removal of the blocking buffer, 100 μL PBST was added to each well and mixed thoroughly. A 20 μL aliquot from each well was transferred to a black 384-well flat-bottom plate (Corning, 3820) for background (blank) measurements.

The remaining PBST was removed, and the beads were incubated with SA-647 (500 nM in 1:1 PBST/blocking buffer, 150 μL per well) for 1 h at room temperature. After incubation, wells were washed with PBST while shaking (3 × 20 min). A final 150 μL PBST wash was performed and drained. Fresh PBST (80 μL) was added to each well, and the contents were transferred to labeled PCR tubes. Samples were briefly vortexed and mixed by pipetting.

Each sample was transferred to a black 384-well plate in triplicate (3 × 20 μL per well). The plate was sealed with a foil sticker and centrifuged at 250*g* for 1 min. Fluorescence intensity was measured using a TECAN plate reader. Background fluorescence (from blank wells) was subtracted prior to further data analysis.

### Competition Assay with Biotin

This assay was conducted following the exact same procedure described above using two samples: SA-647 (500 nM) alone, and SA-647 (500 nM) that had been pretreated with biotin (10 μM) for 30 min at room temperature. Each sample was added to separate wells of a microtiter plate.

#### Screening Using Multiscreen Plates for GST-Rpan13 PRU and GST

General notes:

- At each step, contents were mixed by pipetting up and down 3–5 times.
- All steps following the addition of Alexa Fluor 647 anti-FLAG Tag Antibody were conducted in the dark.
- Alexa Fluor 647 anti-FLAG Tag Antibody was purchased from commercial sources; (BioLengend cat #637316).

A 96-well Multiscreen hydrophilic assay plate (MilliporeSigma, MSBVN12) was activated by treating each well with 150 μL of 70% (v/v) ethanol in water. The solvent was removed under vacuum, and the wells were washed with PBST (3 × 150 μL). Fresh PBST (150 μL) was then added to each well.

Bead-displayed compounds (∼1 × 10^6^ beads in 100 μL DMF) were added individually to separate wells (one compound per well). The plate was drained and washed again with PBST (3 × 150 μL), followed by the addition of 150 μL PBST per well. Plates were shaken overnight at ambient temperature on an orbital plate shaker.

The next day, wells were drained and incubated with 150 μL of a 1:1 (v/v) solution of PBST/PBS blocking buffer (Thermo Scientific, 37538) for 1 h at room temperature. After removal of the blocking buffer, 100 μL PBST was added to each well and mixed thoroughly. A 20 μL aliquot from each well was transferred to a black 384-well flat-bottom plate (Corning, 3820) for background (blank) measurements.

The remaining PBST was removed, and the beads were incubated with GST-Rpn13 PRU or GST (500 nM in 1:1 PBST/blocking buffer, 150 μL per well) for 1 h at room temperature. After incubation, wells were washed with PBST while shaking (3 × 20 min). A final 150 μL PBST wash was performed and drained. Then the beads were incubated with Alexa Fluor 647 anti-FLAG Tag Antibody (5 nM in 1:1 PBST/blocking buffer, 150 μL per well) for 1 h at room temperature. After incubation, wells were washed with PBST (150 μL per well, 5×). Fresh PBST (80 μL) was added to each well, and the contents were transferred to labeled PCR tubes. Samples were briefly vortexed and mixed by pipetting.

Each sample was transferred to a black 384-well plate in triplicate (3 × 20 μL per well). The plate was sealed with a foil sticker and centrifuged at 250*g* for 1 min. Fluorescence intensity was measured using a TECAN plate reader. Background fluorescence (from blank wells) was subtracted before further data analysis.

#### Competition Assay with Rpn2 Peptide for GST-Rpn13 PRU Fusion Protein

This assay was conducted following the exact same procedure described above using two samples: GST-Rpn13 PRU (100 nM) alone, and GST-Rpn13 PRU (100 nM) that had been pretreated with Rpn2 peptide (100 μM) for 1 h at 4 °C. Each sample was added to separate wells of a microtiter plate.

#### Competition Assay with Glutathione for GST-Rpn13 Pru Fusion Protein

This assay was conducted following the exact same procedure described for screening, using GST-Rpn13 PRU fusion protein (500 nM) that had been pretreated with glutathione (1 mM) for 1 h at 4 °C. Each sample was added to separate wells of a microtiter plate.

#### Mock HTS Screening by FACS

General notes:

- At each step, contents were mixed by pipetting up and down 3–5 times.
- All steps following the addition of SA-647 were conducted in the dark.

Approximately 25,000 beads from each mock library compound, control fragments (2, 3), the oxazole analog of 2, and blank beads (∼1.3 × 10^6^ total beads) were added to each of two Mobicol columns equipped with large-pore frits (10 μm; MoBiTec M1003 and M2210-P) in DMF. The beads were washed with PBST (3 × 300 μL) and equilibrated in PBST overnight (18 h) at room temperature with gentle rotation.

The following day, the PBST was drained, and each column was incubated with 150 μL of a 1:1 (v/v) solution of PBST/PBS blocking buffer (Thermo Scientific, 37538) for 1 h at room temperature. After removal of the blocking buffer, one Mobicol column was treated with SA-647 (500 nM in 1:1 PBST/blocking buffer, 300 μL) for 1 h at room temperature. The second column served as a no-protein control.

Following incubation, beads were washed with PBST (3 × 300 μL, 20 min each) with gentle agitation, followed by a final 300 μL wash and drainage. Beads were resuspended in 500 μL PBST and passed through a 35 μm cell strainer into FACS tubes (Falcon). Sorting was performed using a BD FACS Fusion cell sorter, collecting populations that shifted distinctly from the double-negative control in the red fluorescence channels.

Data were analyzed using FlowJo Software (v10.6.1; Becton, Dickinson and Company, Ashland, OR; 2019).

#### Click Reaction to Install the Flight-Tag for Sorted Hits

Sorted hits (approximately 37000 beads) from the FACS screen were collected in a Mobicol, and the collection buffer was drained. DMF (300 μL) was added and incubated for 10 min to reswell the beads and to denature the bound protein. After washing the beads with DMF (300 μL, 2×) beads were washed with PBST (300 μL, 3×).

To generate the catalyst cocktail for the CuAAC (“click”) reaction, a 100 mM solution of sodium ascorbate was freshly prepared in deionized water. Separately, 100 mM stock solutions of copper­(II) sulfate (CuSO_4_) and the ligand THPTA were prepared in water. The catalyst mixture was assembled by combining 70 μL of water, 25 μL of the sodium ascorbate solution, 1 μL of the CuSO_4_ solution, and 4 μL of the THPTA solution.

For the conjugation step, 75 μL of a 0.5 M solution of the flight-Tag (in 1:1 DMSO/H_2_O) was added directly to the sorted hit beads following their washing steps. Then, 165 μL of water, 30 μL of the freshly prepared catalyst cocktail, and 30 μL of 10× phosphate-buffered saline (PBS, pH 7.0) were added. The suspension was briefly vortexed to ensure complete mixing and incubated at 37 °C for 1 h with gentle shaking.

After the reaction, the coupling solution was removed, and the beads were washed with PBST (300 μL, 3×). To remove residual copper, the beads were treated twice (30 min each) with a pH 7 aqueous buffer containing 6 M guanidinium chloride, 0.1 M EDTA, and 0.1 M sodium dihydrogen phosphate. The beads were subsequently washed with water (3×), DMF (3×), and DCM (3×). Finally, the thoroughly washed beads were transferred into a 600 μL Eppendorf tube in a 3:1 mixture of DCM/MeOH (400 μL).

#### Photocleavage and Hit Identification in LC-MS

The beads suspended in the 3:1 DCM/MeOH mixture were irradiated at 365 nm (4.5 × 10^3^ μJ cm^–2^) for 1 h using a UVP CL-1000L UV cross-linker to release the ligands from the solid support. Following photocleavage, the solvents were evaporated using a heating block. The resulting residue was redissolved in 400 μL of a 1:1 methanol/water mixture and filtered through a 0.45 μm PVDF syringe filter into 1.5 mL Eppendorf tubes. The filtrates were snap-frozen and lyophilized overnight to remove residual solvents. The lyophilized material was then reconstituted in 50 μL of Optima-grade water and analyzed by LC-MS to identify potential hit compounds.

Note: Photo released hits are expected to exhibit a distinct isotopic signature due to the presence of bromine from the Flight-Tag conjugate.

#### *Z*′ Factor Calculation

*Z*′-factor was calculated following the same multiscreen plate method described above. Compound 2 and Compound 3 displayed beads were used as controls, with 12 replicates of each compound processed in separate wells following the identical screening protocol.

For *Z*′ factor determination, compound 3 served as the negative control and compound 2 as the positive control. Each compound was processed through the complete multiscreen assay workflow: bead activation, blocking, SA-647 incubation (500 nM), washing steps, and fluorescence measurement using the TECAN plate reader. Background fluorescence from blank wells was subtracted from all measurements prior to *Z*′ score calculation.

The *Z*′ factor was calculated using the formula^35^

Where

$$Z^{\prime }=1-\frac{\left({3\sigma }_{c+}\right)+\left({3\sigma }_{c-}\right)}{\left|\mu_{c+}-\mu_{c-}\right|}$$

**σ**_**c+**_ = Standard deviation of fragment 2 (positive control, *n* = 12).

**σ**_**c–**_ = Standard deviation of fragment 3 (negative control, *n* = 12).

**μ**_**c+**_ = Mean fluorescence intensity of fragment 2 (positive control, *n* = 12).

**μ**_**c–**_ = Mean fluorescence intensity of fragment 3 (negative control, *n* = 12).

Table S1 (see SI) shows the individual data for each replicate along with the calculated mean and standard deviation. Using these values in the equation above gives a *Z*′ score of 0.66.

## Supplementary Material





## Notes

Supporting Figures, Solid-phase synthetic Schemes, LC traces of all compounds, and NMR spectra of compounds (PDF)
SMILES strings and solution dissociation constants of compounds (where applicable) (CSV)

## Abbreviations Used

**Avi**: avi biotin-acceptor tag
**CM5**: carboxymethylated dextran sensor chip
**CuAAC**: copper-catalyzed azide–alkyne cycloaddition
**CuSO**_**4**_: copper­(II) sulfate
**DCM**: dichloromethane
**DIC**: *N*,*N*′-diisopropylcarbodiimide
**DIPEA**: *N*,*N*-diisopropylethylamine
**DMF**: *N*,*N*-dimethylformamide
**DMSO**: dimethyl sulfoxide
**DTT**: dithiothreitol
**EDTA**: ethylenediaminetetraacetic acid
**ESI**: electrospray ionization
**FACS**: fluorescence-activated cell sorting
**FBDD**: fragment-based drug discovery
**Fmoc**: 9-fluorenylmethyloxycarbonyl
**GST**: glutathione S-transferase
**HBS**: HEPES-buffered saline
**HBS–TE**: HEPES-buffered saline with TCEP and EDTA
**HPLC**: high-performance liquid chromatography
**HRMS**: high-resolution mass spectrometry
**HTS**: high-throughput screening
**IPTG**: isopropyl β-d-1-thiogalactopyranoside
***K***_**D**_: dissociation constant
**LC-MS**: liquid chromatography–mass spectrometry
**LB**: Luria–Bertani medium
**MeOH**: methanol
**mSA**: monomeric streptavidin
**MWCO**: molecular weight cutoff
**NMR**: nuclear magnetic resonance
**Ni–NTA**: nickel–nitrilotriacetic acid
**OD**_**600**_: optical density at 600 nm
**Oxyma**: ethyl (hydroxyimino)­cyanoacetate
**PBS**: phosphate-buffered saline
**PBST**: phosphate-buffered saline with Tween-20
**PD-10**: sephadex G-25 desalting column
**PMSF**: phenylmethylsulfonyl fluoride
**PVDF**: poly­(vinylidene difluoride)
**Rpn13**: regulatory particle non-ATPase 13
**RU**: response unit
**RT**: room temperature
**SA**: streptavidin
**SA-647**: streptavidin conjugated to alexa fluor 647
**SDS-PAGE**: sodium dodecyl sulfate–polyacrylamide gel electrophoresis
**SD**: standard deviation
**SPR**: surface plasmon resonance
**TCEP**: tris­(2-carboxyethyl)­phosphine
**TFA**: trifluoroacetic acid
**THPTA**: tris­(3-hydroxypropyltriazolylmethyl)­amine
**TIPS**: triisopropylsilane
**WT**: wild type
