# Supplementary material for: Exploiting Avidity Effects for the Discovery of Low Affinity Protein-Binding Fragments
Source: J Med Chem. 2025 Sep 15;68(18):19521–35. doi: 10.1021/acs.jmedchem.5c01742 (PMC12598851; doi:10.1021/acs.jmedchem.5c01742)
Supplement: Supplementary file 1 [file jm5c01742_si_001.pdf]

Supporting Information

Exploiting Avidity Effects for the Discovery of Low Affinity Protein-Binding Fragments

Isuru M. Jayalath,[†] Donella Beckwith,[ƒ] Jihyeon Yoon,[∂] Xingui Liu,[∂], and Thomas Kodadek[†]*

[†]Department of Chemistry, The Herbert Wertheim UF Scripps Institute for Biomedical Innovation & Technology, 120 Scripps Way, Jupiter, FL 33458 USA.

[ƒ] Deluge Biotechnologies, 6671 W. Indiantown Rd. Suite 50-325, Jupiter, FL 33458 USA.

[∂]Department of Medicinal Chemistry, College of Pharmacy, University of Florida, Gainesville, FL 32610 USA.

*To whom correspondence should be addressed: kodadek@ufl.edu

Table of Contents

Part I: Supporting Figures

Figure S1. SPR analysis of compound 3 binding to immobilized streptavidin S4

Figure S2. Competition of compound 2-displayed beads with biotin S4

Figure S3. Biotin-displayed 10 μm TentaGel beads S5

Figure S4. Oxazole version of compound 2 displayed 10 μm TentaGel beads S5

Figure S5. Structures of carboxylic acids used for acylation in compound growth experiments S6

Figure S6. SPR analysis of compound 3 binding to immobilized streptavidin S7

Figure S7. SPR analysis of compound 3 binding to immobilized streptavidin S7

Figure S8. SPR analysis of compound 6 binding to immobilized streptavidin S8

Figure S9. SPR analysis of compound 7 binding to immobilized streptavidin S8

Figure S10. Structures of carboxylic acids used to generate a 94-compound bead-displayed library S9

Figure S11. Binding experiment between Rpn13 PRU hit–, Rpn2-displayed beads, and acetylated beads with FLAG antibody.. S10

Figure S12. SPR analysis of compound A63 binding to immobilized Rpn13 PRU S10

Figure S13. Structures of carboxylic acids used to generate a 51-compound bead-displayed library for pooled screening.. S11

Part II: Solid-Phase Synthetic Schemes and Characterization Data

Scheme 1. Solid-phase synthesis of bead-displayed compound 2 .... S12

Scheme 2. Solid-phase synthesis of bead-displayed compound 3 .... S12

Scheme 3. Solid-phase synthesis of oxazole version of compound 2 .... S13

Scheme 4. Solid-phase extension from bead-displayed compound 8 .... S13

Scheme 5. On-bead synthesis of compound 9 and mock library for FACS S13

Figure S14. LC trace of purified mini-PEG attached compound 2 .... S14

Figure S15. ^{1}H NMR spectrum of mini-PEG attached compound 2 at 22.4 °C S14

Figure S16. ^{1}H NMR spectrum of mini-PEG attached compound 2 at 100 °C S15

Figure S17. ^{13}C{^{1}H} NMR spectrum of mini-PEG attached compound 2 .... S15

Figure S18. LC trace of purified mini-PEG attached compound 3 .... S16
Figure S19. LC trace of purified mini-PEG attached compound 4 .... S16
Figure S20. LC trace of purified mini-PEG attached compound 5 .... S17
Figure S21. LC trace of purified mini-PEG attached compound 6 .... S17
Figure S22. LC trace of purified mini-PEG attached compound 7 .... S18
Figure S23. LC trace of purified mini-PEG attached compound A63 .... S18
Figure S24. LC trace of purified mini-PEG attached compound A85 .... S19
Figure S25. LC trace of purified Rpn2 peptide S19
Figure S26. ^{1}H NMR spectrum of compound 10 in DMSO-d_6 .... S20
Figure S27. $^{13}C\{^{1}H\}$ NMR spectrum of compound 10 in DMSO-d_6 .... S20
Table S1. Fluorescence intensity data for Z' calculation S21

Part I: Supporting Figures

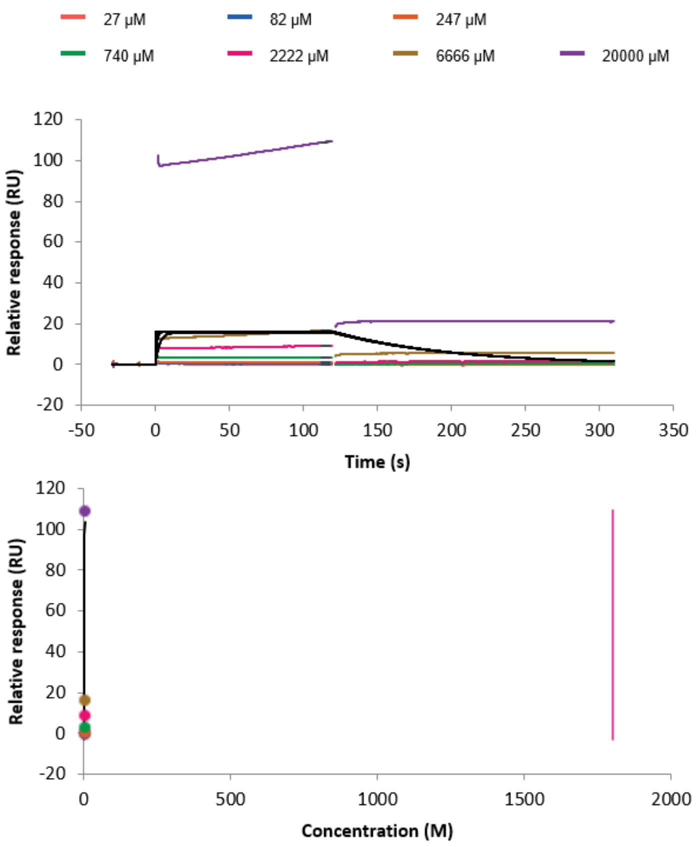

Figure S1: SPR analysis of compound 3 binding to immobilized streptavidin.

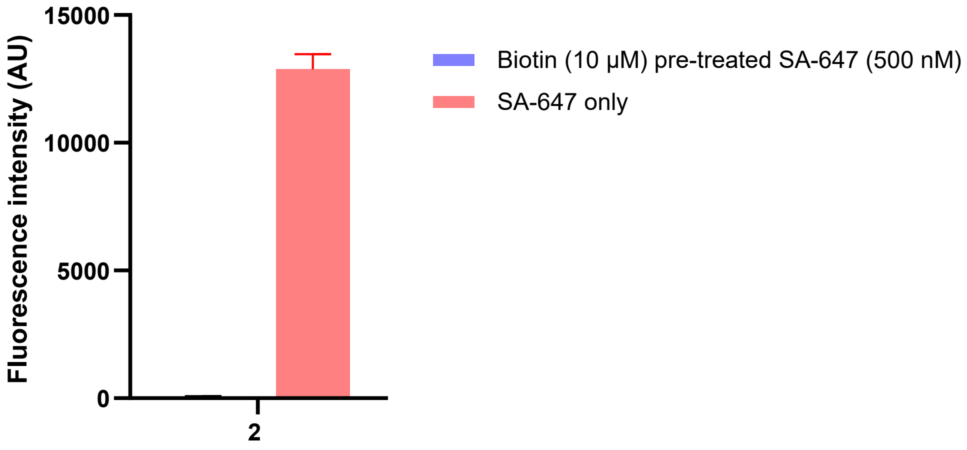

Figure S2: Competition of compound 2-displayed beads with biotin. (Access biotin pre-treated SA-647 was incubated with ligand displayed beads).

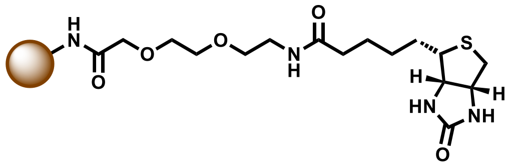

Figure S3: Biotin-displayed 10 μm TentaGel beads.

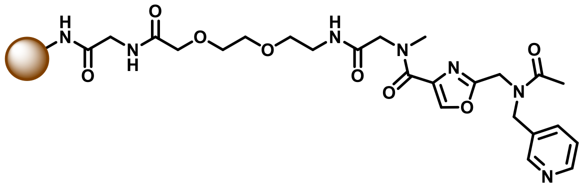

Figure S4: Oxazole version of compound 2 displayed 10 μm TentaGel beads.

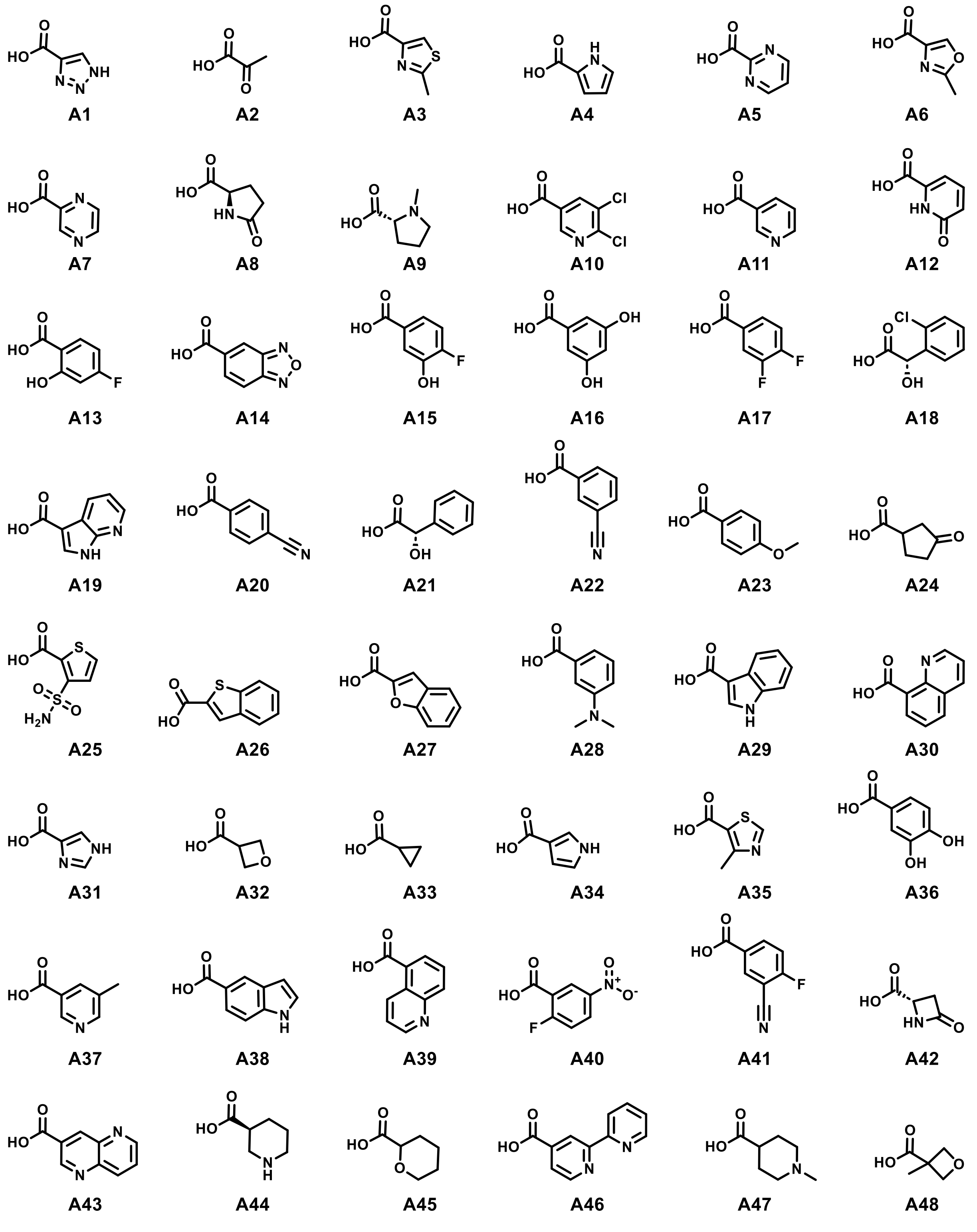

Figure S5: Structures of carboxylic acids used for acylation in compound growth experiments.

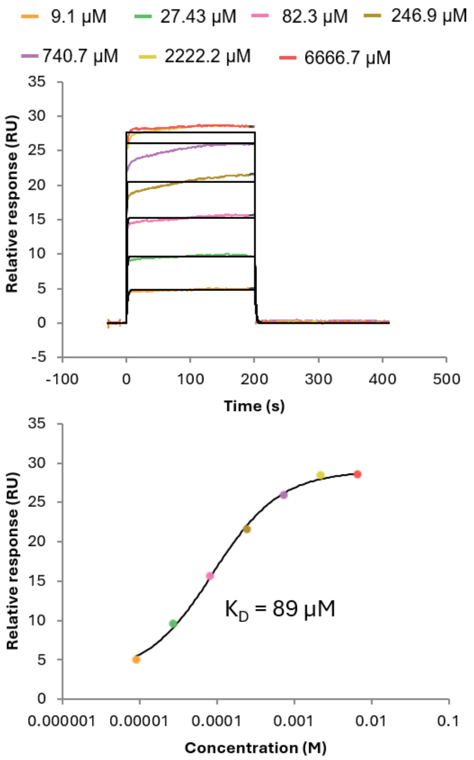

Figure S6: SPR analysis of compound 3 binding to immobilized streptavidin.

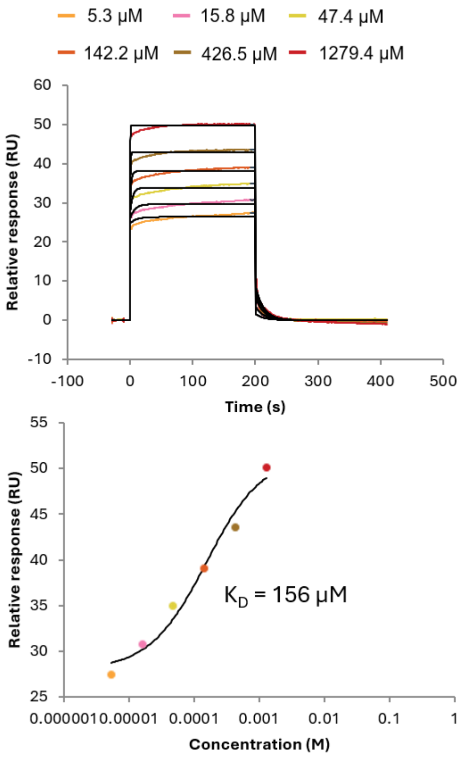

Figure S7: SPR analysis of compound 3 binding to immobilized streptavidin.

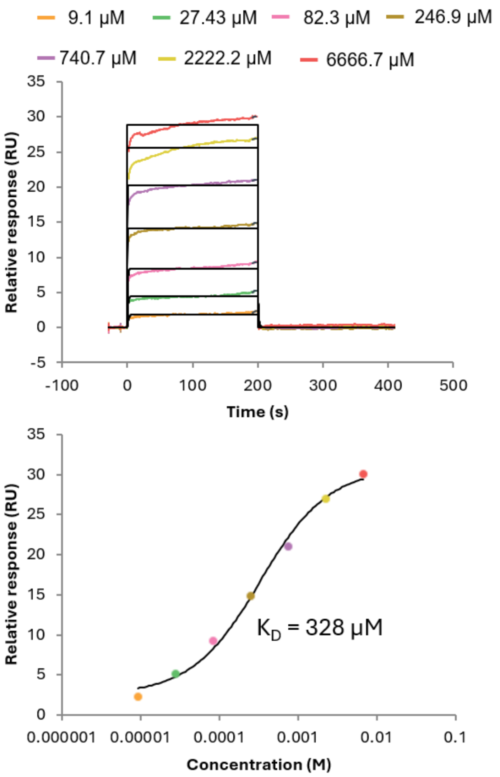

Figure S8: SPR analysis of compound 6 binding to immobilized streptavidin.

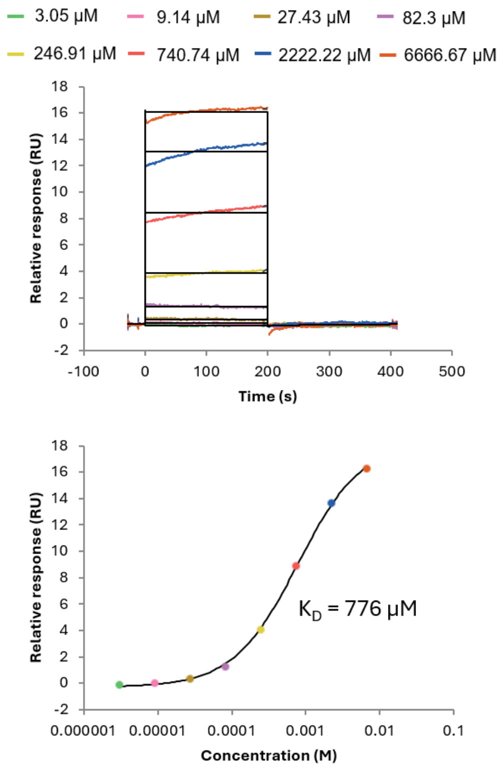

Figure S9: SPR analysis of compound 7 binding to immobilized streptavidin.

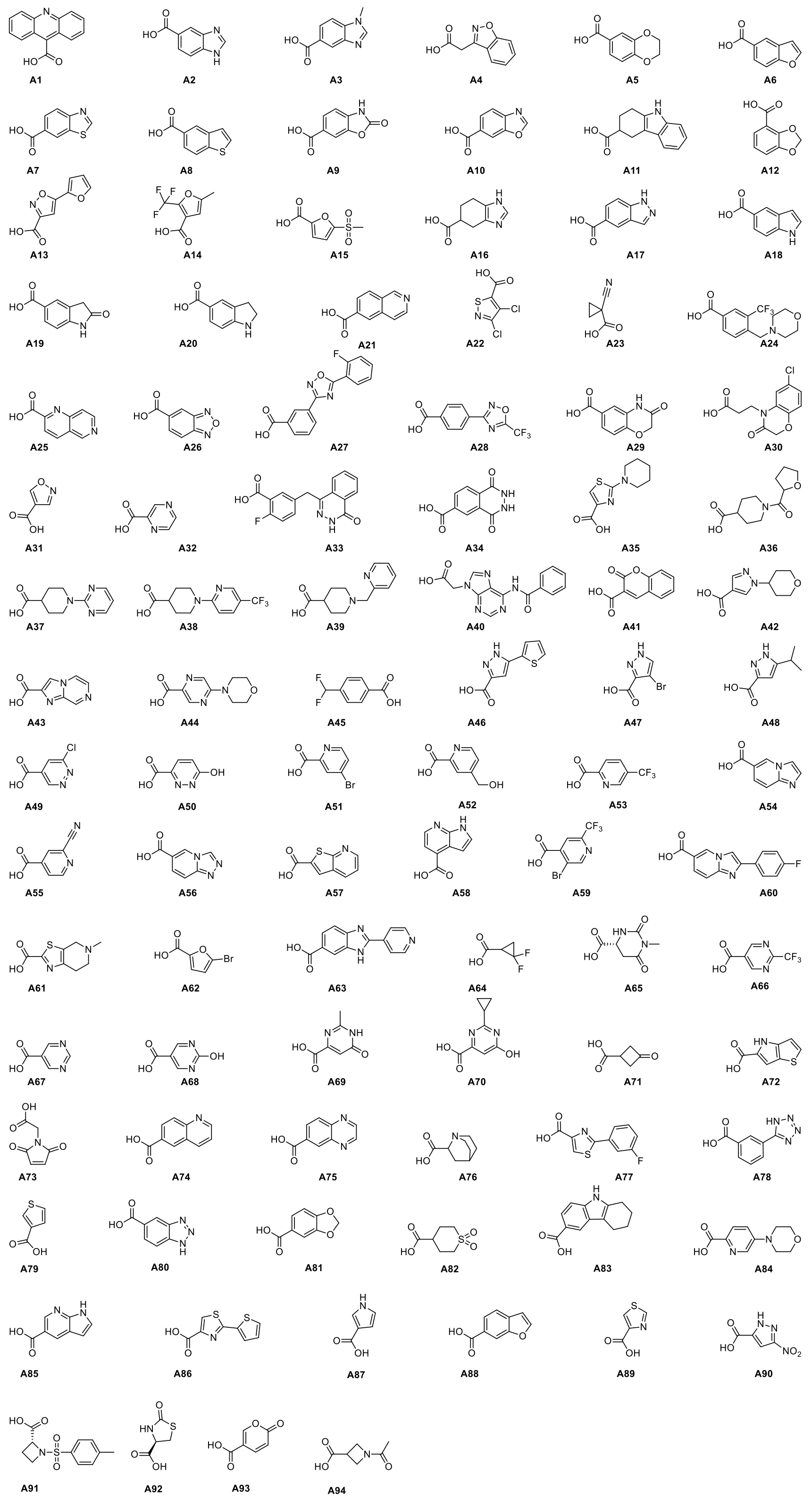

Figure S10: Structures of the 94-carboxylic acid used to acylate the TentaGel beads, generating a small panel of bead-displayed fragments for the screening with GST-Rpn13 fusion protein.

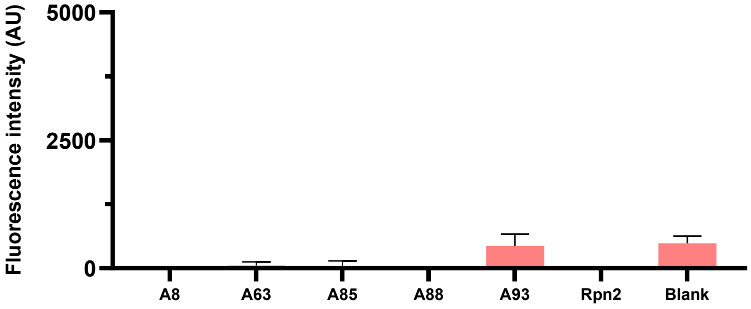

Figure S11: Binding experiment between Rpn13 PRU hit-, Rpn2-displayed beads, and acetylated beads with A-647 FLAG antibody.

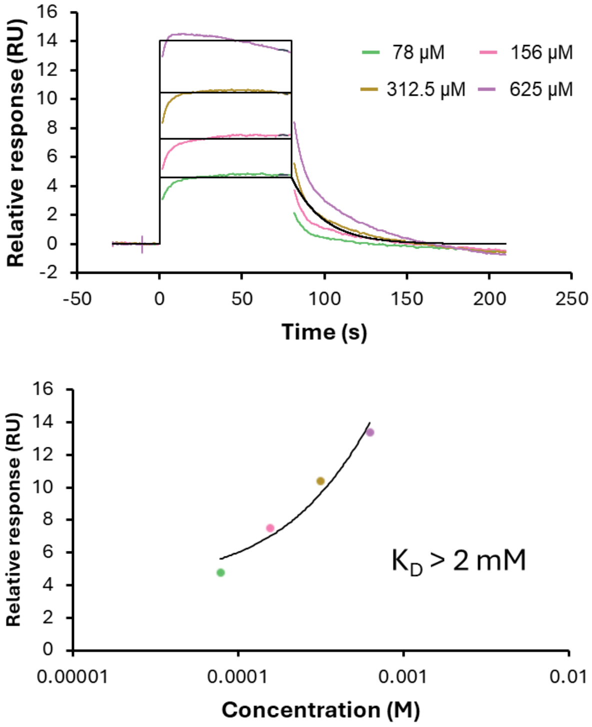

Figure S12: SPR analysis of compound A63 binding to immobilized Rpn13 PRU domain. (Immobilized via biotinylated Avi-Tag on SA chip)

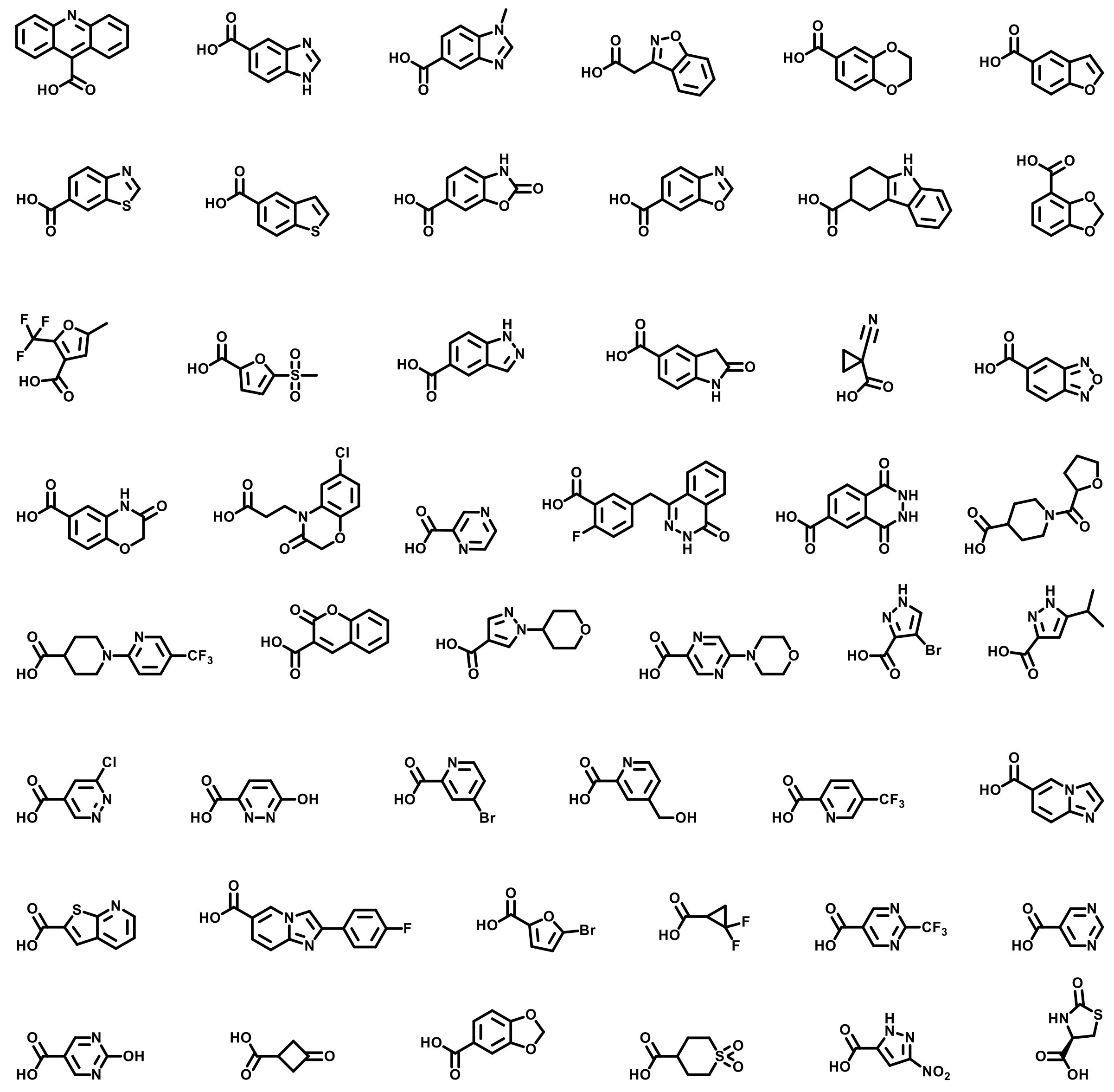

Figure S13: Structures of carboxylic acids used to generate a bead-displayed 51-compound library to screen against SA in the pooled FACS method.

Part 2: Solid-phase synthetic schemes and characterization data

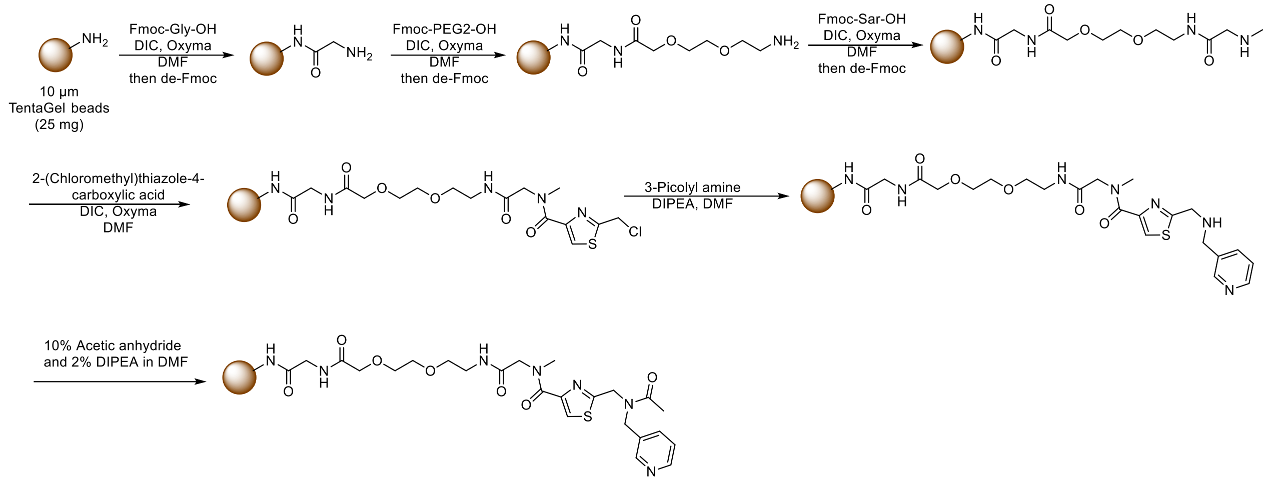

Scheme 1: Solid-phase synthesis of bead-displayed compound 2

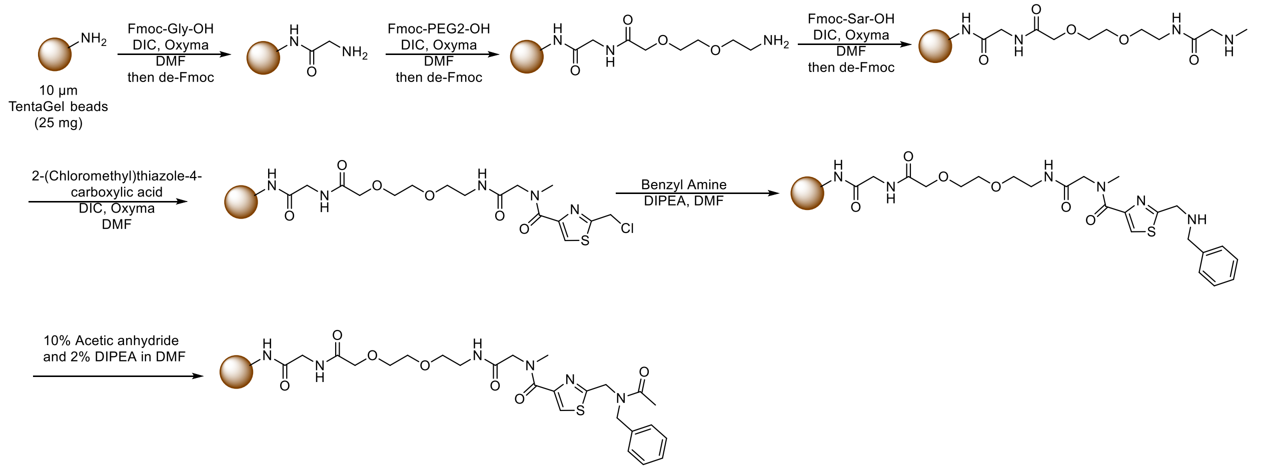

Scheme 2: Solid-phase synthesis of bead-displayed compound 3

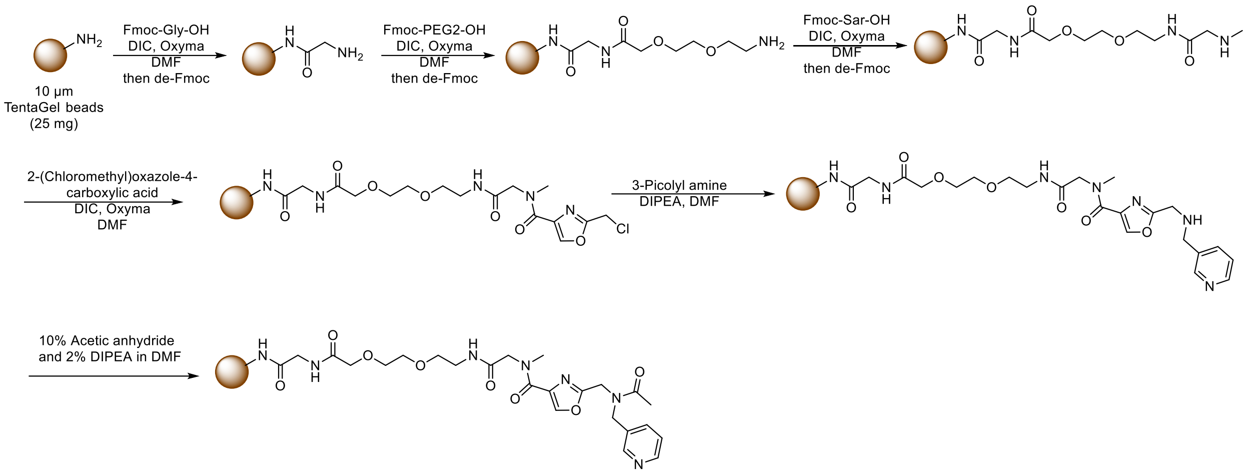

Scheme 3: Solid-phase synthesis of the bead-displayed oxazole version of compound 2

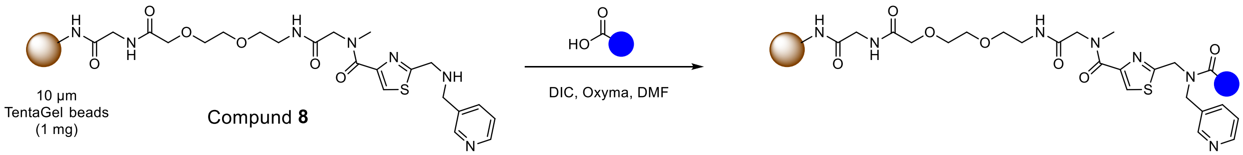

Scheme 4: Solid-phase compound extension starting from bead-displayed compound **8**

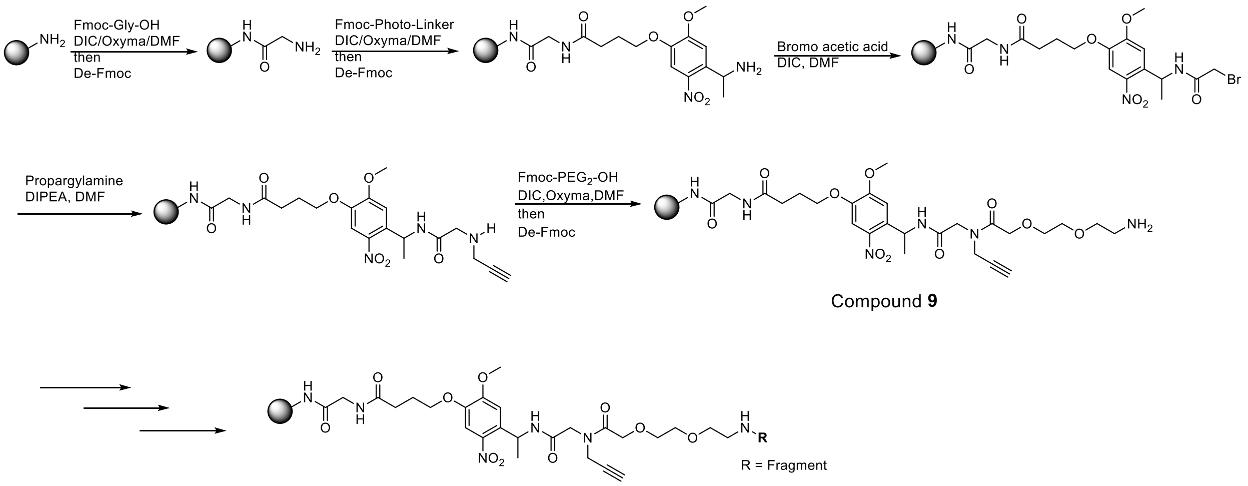

Scheme 5: On-bead synthesis of compound **9** and on-bead mock library synthesis for pooled FACS sorting experiment.

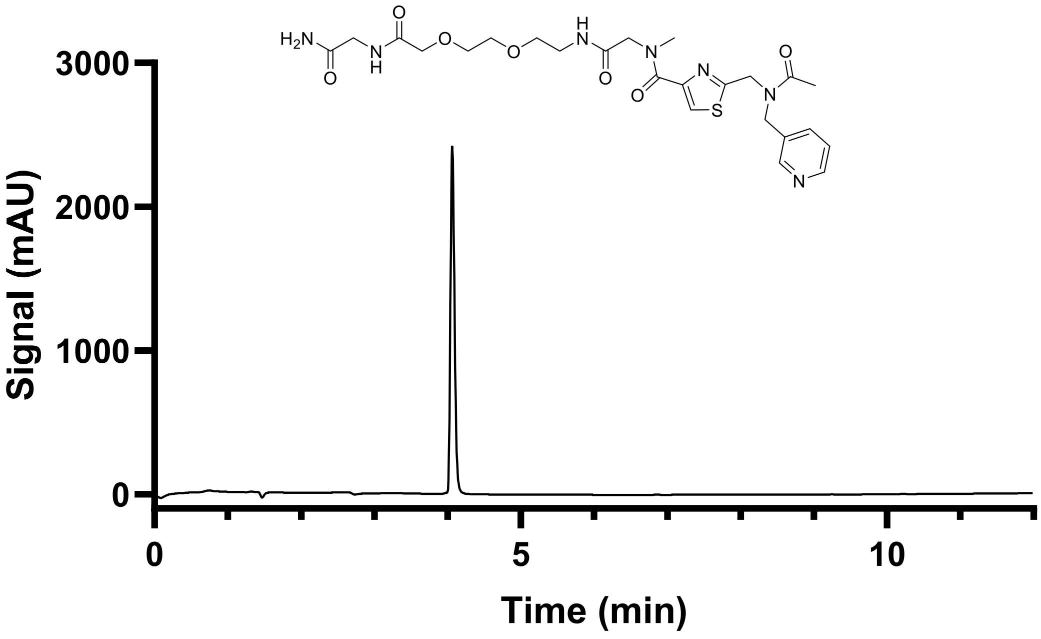

Figure S14: LC trace of the purified mini-PEG attached compound 2.

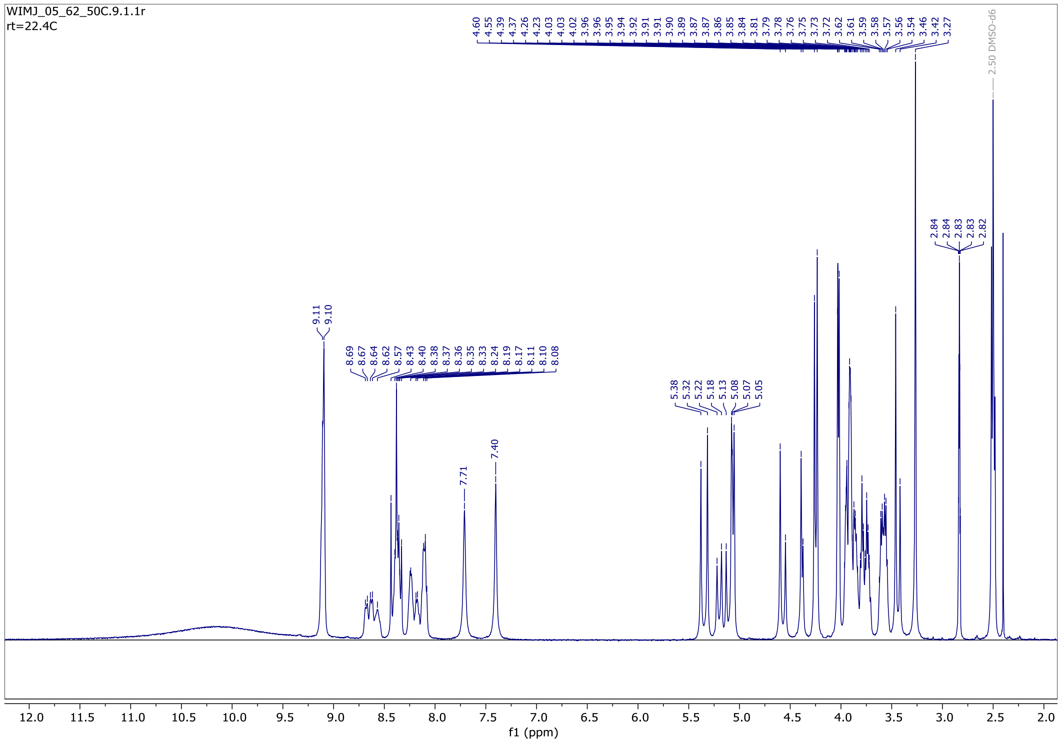

Figure S15: ^{1}H NMR spectrum of mini-PEG attached compound 2 at room temperature (22.4 °C) in DMSO-d_6.

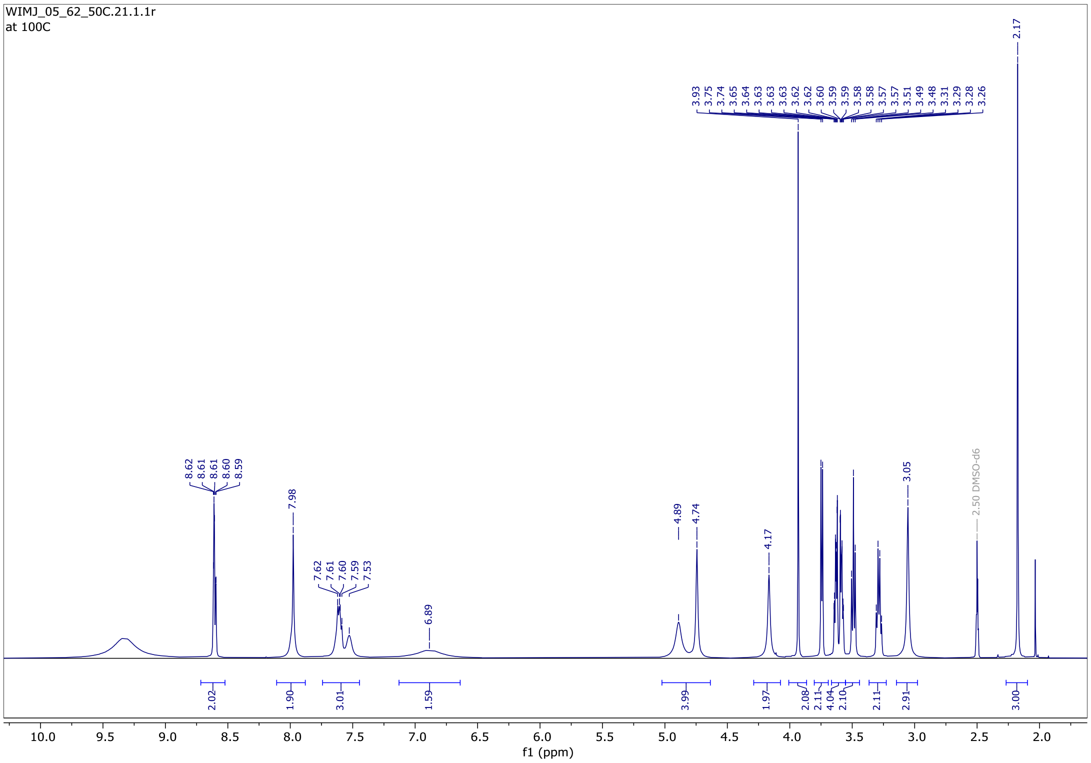

Figure S16: ^{1}H NMR spectrum of mini-PEG attached compound 2 at 100 °C in DMSO-d_6.

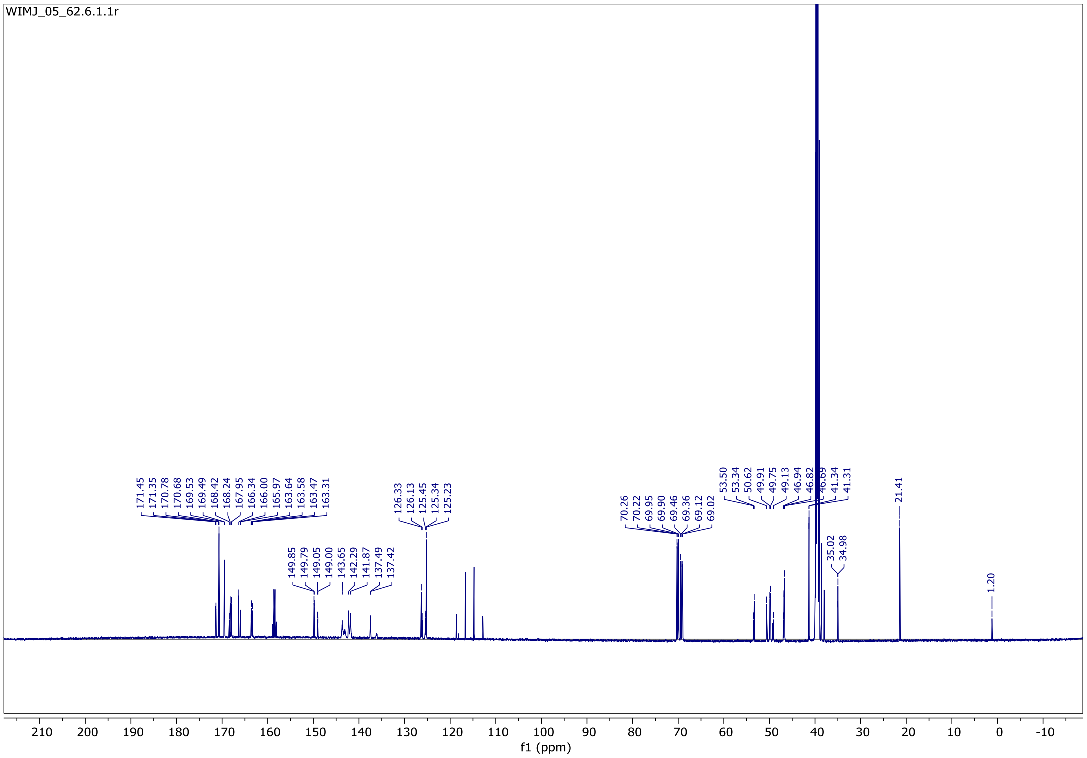

Figure S17: ^{13}C {^{1}H} NMR spectrum (151 MHz) of mini-PEG attached compound 2at room temperature in DMSO-d_6.

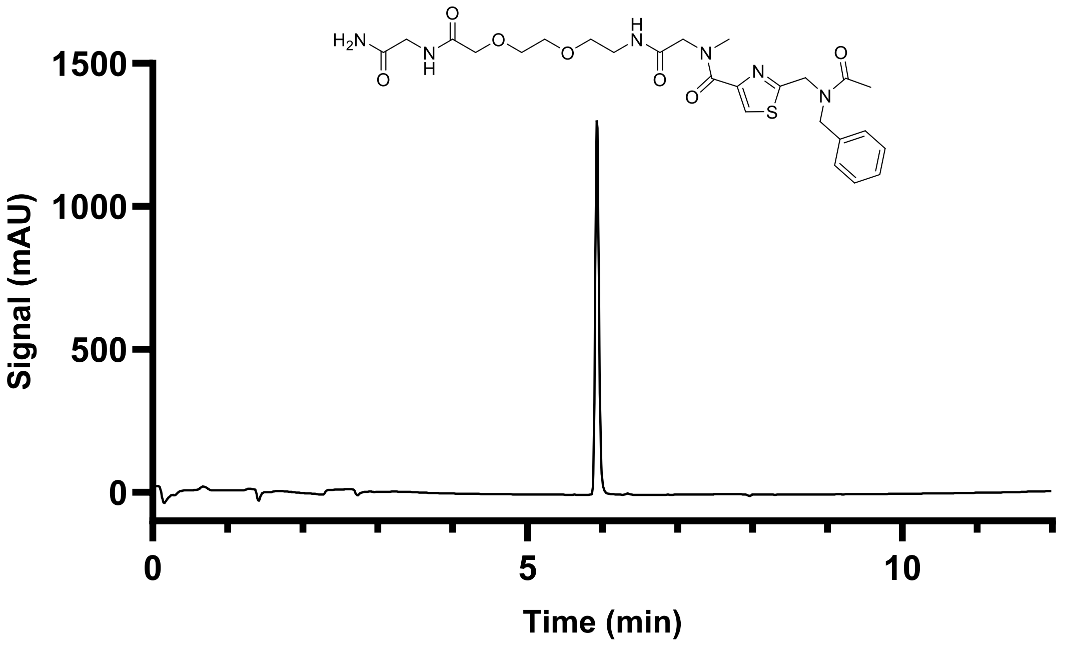

Figure S18: LC trace of the purified mini-PEG attached compound 3.

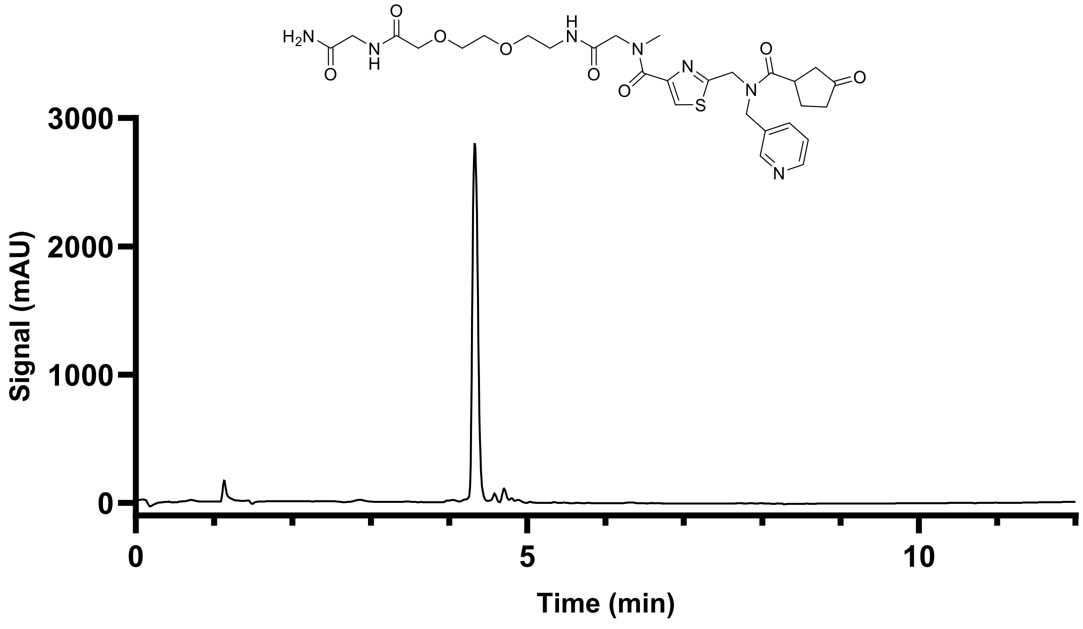

Figure S19: LC trace of the purified mini-PEG attached compound 4.

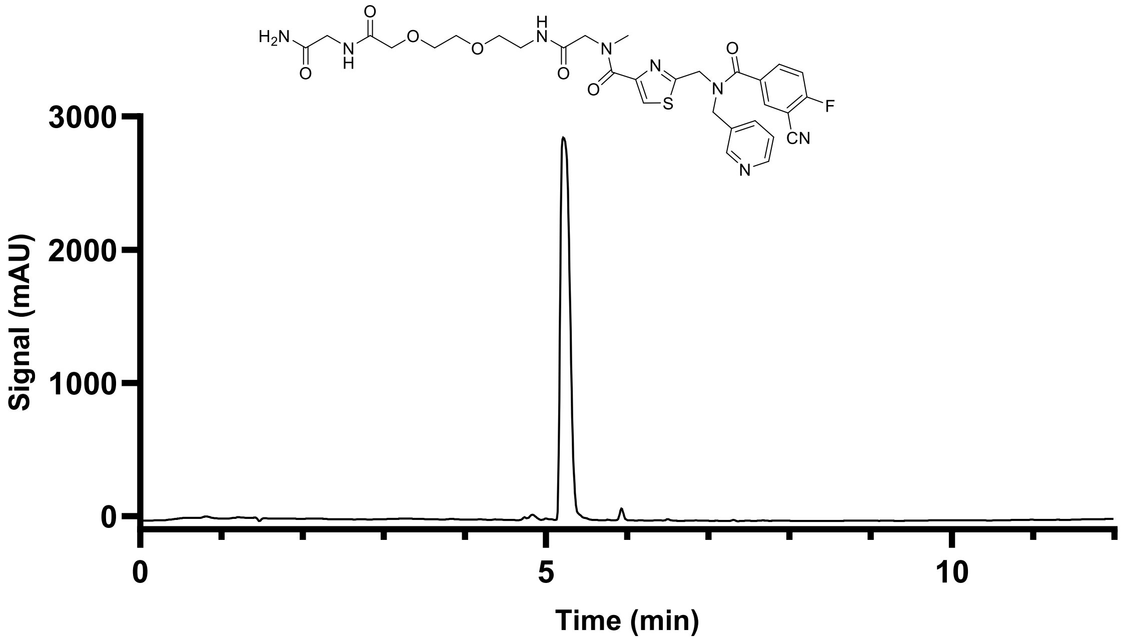

Figure S20: LC trace of the purified mini-PEG attached compound 5.

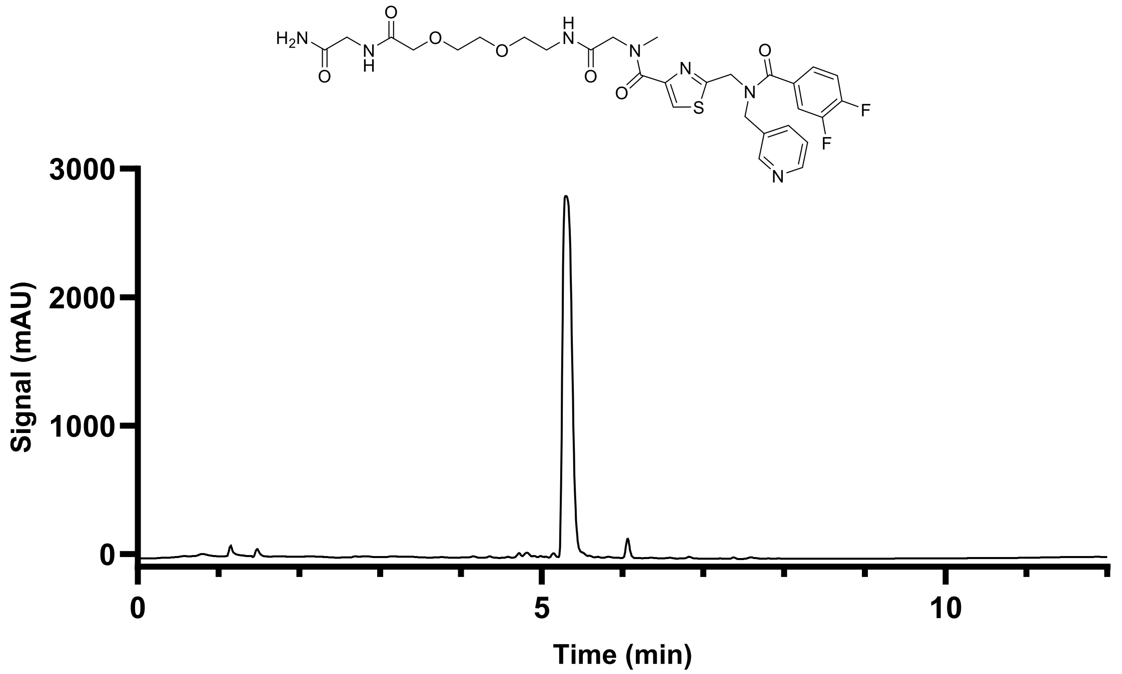

Figure S21: LC trace of the purified mini-PEG attached compound 6.

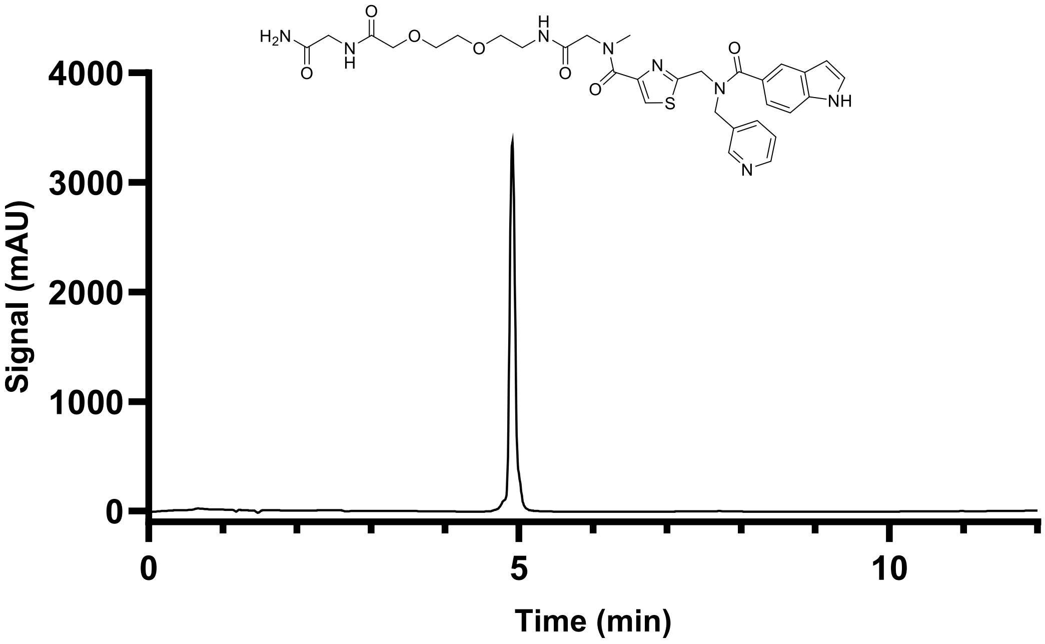

Figure S22: LC trace of the purified mini-PEG attached compound 7.

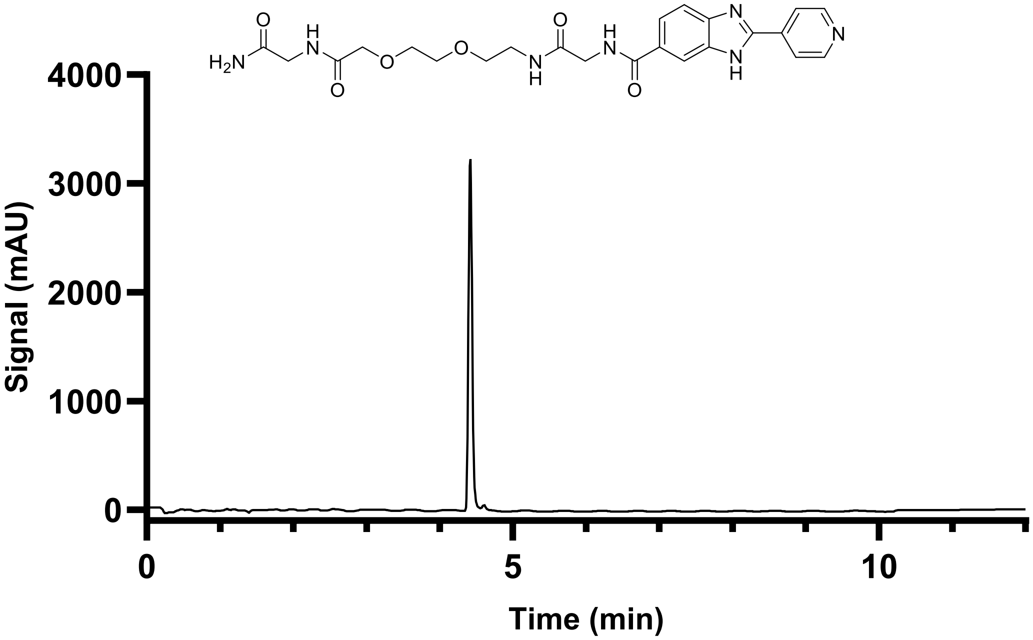

Figure S23: LC trace of the purified mini-PEG attached compound A63.

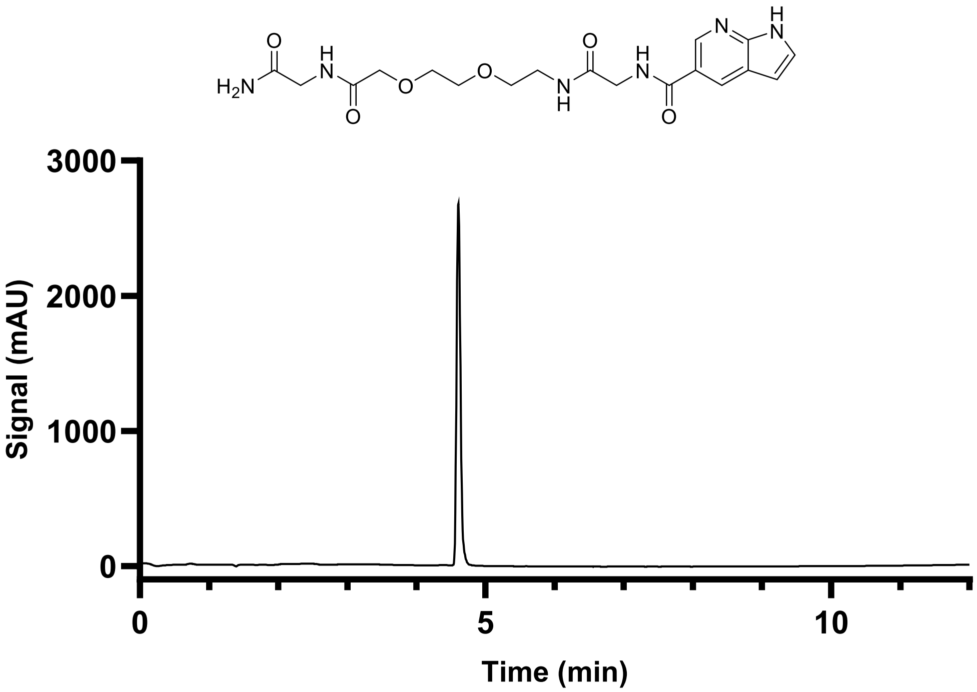

Figure S24: LC trace of the purified mini-PEG attached compound A85.

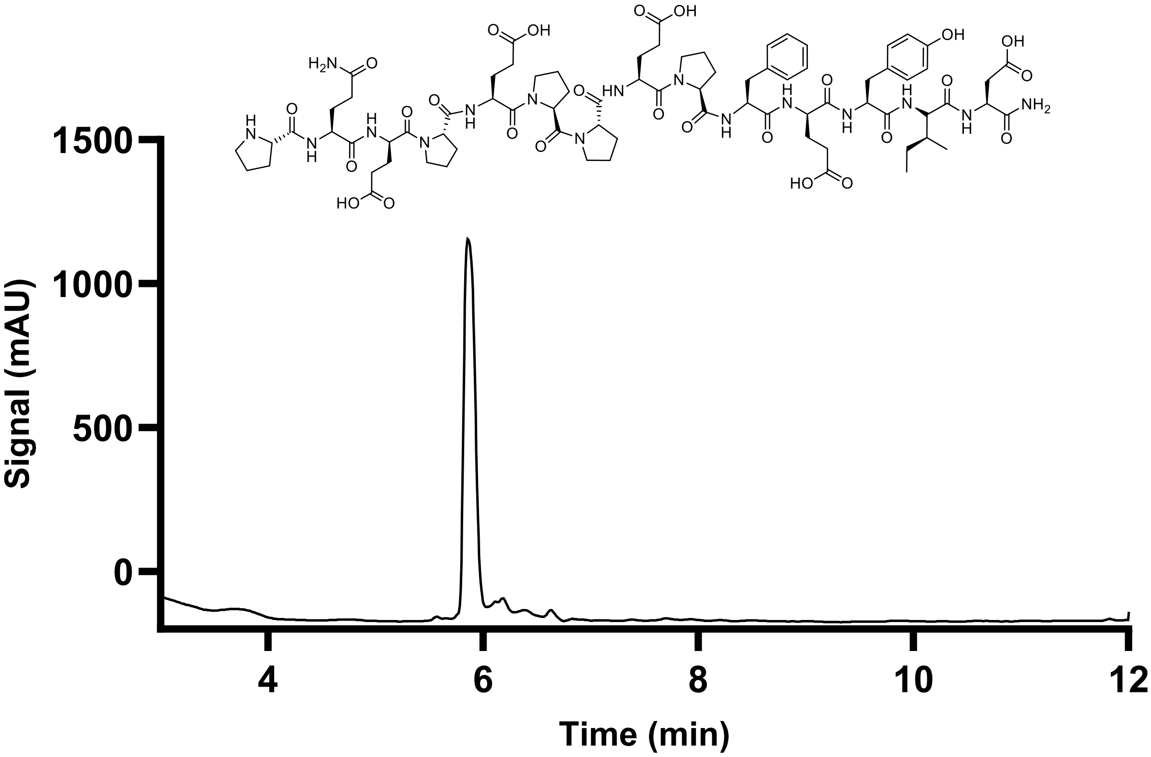

Figure S25: LC trace of the purified Rpn2 peptide.

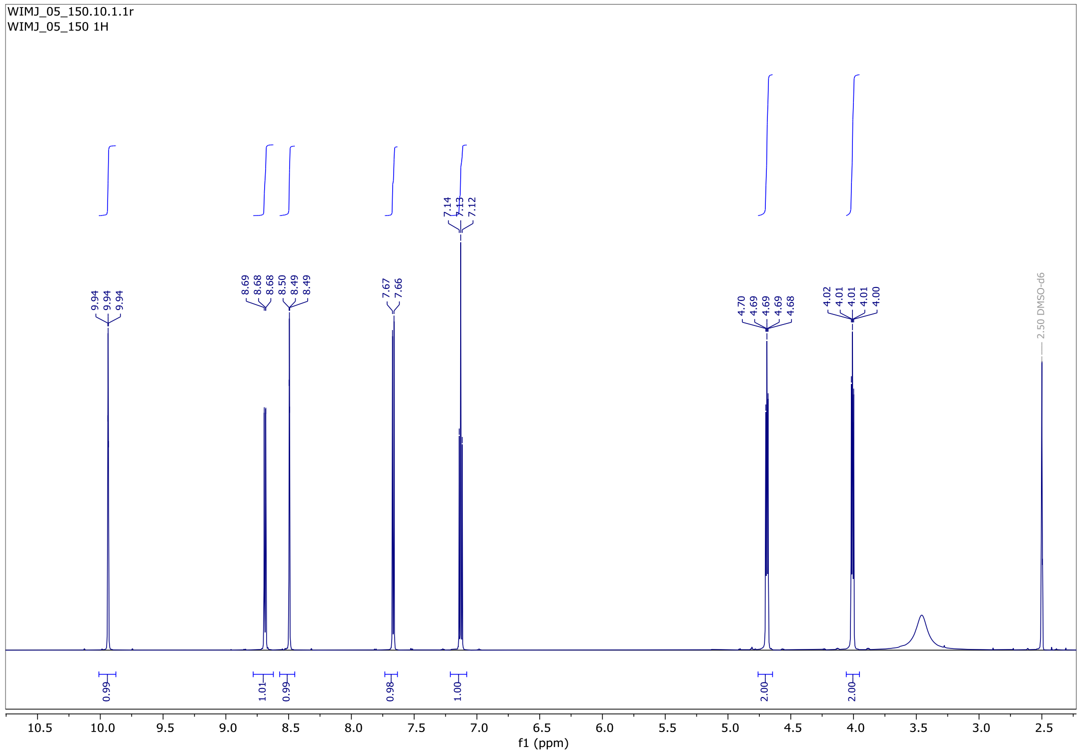

Figure S26: ^{1}H NMR spectrum of compound 10 in DMSO-d_6.

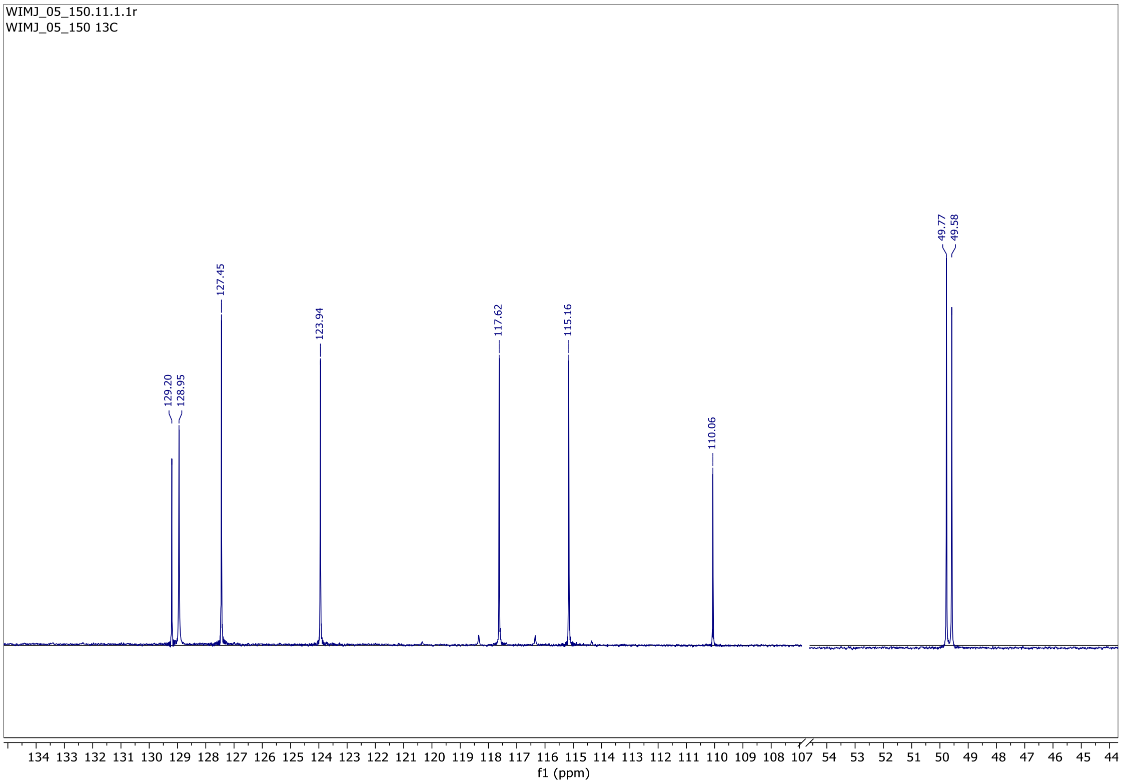

Figure S27: $^{13}C\{^{1}H\}$ NMR spectrum of compound 10 in DMSO-d_6.

Replicate	Fluorescence intensity of compound 2	Fluorescence intensity of compound 3
1	14298	50
2	14769	156
3	15364	122
4	16337	81
5	17794	136
6	14546	180
7	14869	145
8	13797	568
9	15421	76
10	12975	119
11	13104	260
12	12492	226
**Average**	**14647**	**177**
**Standard deviation**	**1496**	**137**

Table S1: Individual fluorescence intensity data for Z' calculation for plate-based assay. (data reported here after background subtraction).
